# Functionalizing Nucleic Acids: Synthesis and Purification Strategies for Bioconjugates as Biomaterials

**DOI:** 10.1002/smll.202510863

**Published:** 2025-12-12

**Authors:** Nico Alleva, Jian Zhang, David Y. W. Ng, Tanja Weil, Torsten John

**Affiliations:** ^1^ Max Planck Institute for Polymer Research Ackermannweg 10 55128 Mainz Germany; ^2^ School of Science Constructor University Campus Ring 1 28759 Bremen Germany

**Keywords:** click chemistry, DNA nanotechnology, hybrid biomaterials, nucleic acid bioconjugates, purification techniques, solid‐phase synthesis, therapeutic delivery

## Abstract

Nucleic acids are fundamental to life, encoding and storing genetic information, catalyzing biological processes, and directing protein synthesis. The primary classes, DNA and RNA, have become increasingly significant in biomedical applications due to advances in stabilizing these structures against degradation and elucidating their molecular roles. Nucleic acids can be functionalized with polymers, peptides, proteins, lipids, saccharides and other functional units to yield hybrid biomaterials with tailored properties for nanomedicine and materials science. This review summarizes conjugation strategies and provides a critical overview of purification approaches, including chromatographic, membrane‐based, and electrophoretic methods, highlighting their principles, advantages, and limitations. Emphasis is placed on the relationship between synthesis route and purification choice, as well as common challenges such as solubility, aggregation, and incomplete coupling. Broadly applicable strategies for the successful synthesis and purification of nucleic acid conjugates are discussed, along with an overview of recent approaches for conjugates with polymers, peptides, proteins, lipids and saccharides. Finally, strategies are summarized for obtaining high‐purity conjugates suitable for biomedical and materials applications.

## Graphical abstract

Nucleic acid‐ligand conjugates combine the distinct properties of macromolecular ligands with the versatility of DNA/RNA nanotechnology. In biomaterial and nanomedicine applications, highly purified products are paramount, requiring reliable synthesis and purification methods. This review provides an overview for obtaining conjugates of nucleic acids with major classes of biomacromolecules, including synthetic polymers, peptides and proteins, lipids, and saccharides.

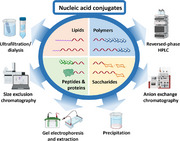

## 1 Introduction

**Figure 1:**
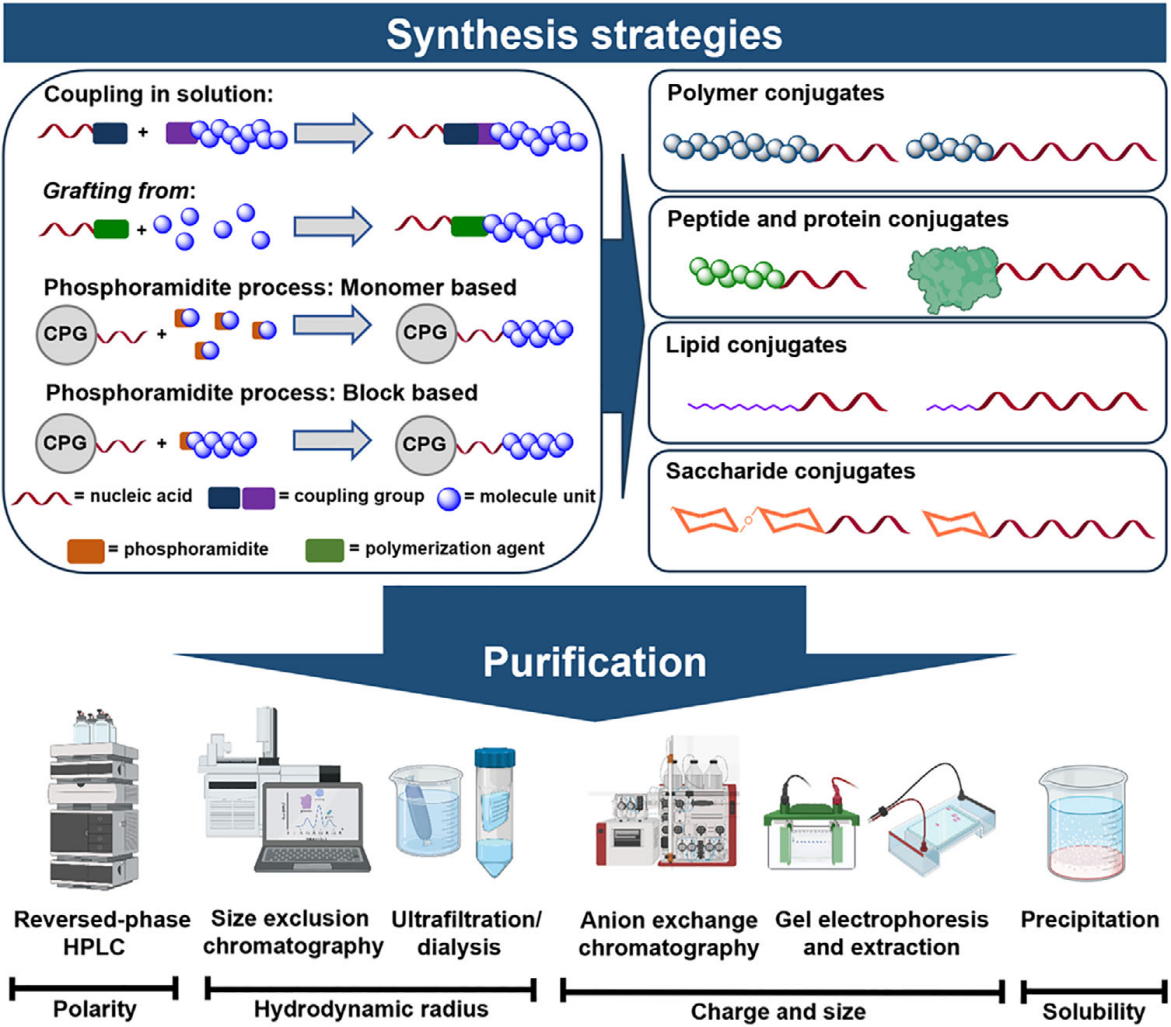
Schematic overview of synthesis and purification strategies of nucleic acid bioconjugates. Focus on conjugates with polymers, peptides and proteins, lipids, and saccharides, along with possible synthesis and purification strategies. CPG = controlled pore glass. (Figure created with BioRender.com).

Nucleic acids are essential building blocks of life; they store genetic information encoded by their sequences and direct cellular processes such as protein synthesis. DNA and RNA fold into characteristic tertiary structures, including the DNA double helix.^[^ ^1^ ^]^ The self‐assembly of nucleic acids into 3D structures is guided by hybridization through hydrogen bonding between complementary nucleobases and stabilized primarily by base stacking and hydrophobic interactions, modulated by electrostatic and conformational factors. The precise hybridization properties and resulting structural diversity of nucleic acids offer unique versatility for nanoscale functionalization, which can be harnessed to develop tailored nanomaterials. The nanoscale addressability of DNA and RNA, along with their complex architectures, make them intriguing scaffolds for materials science. The field known as DNA or RNA nanotechnology involves using nucleic acids to make programmable nanoscale structures used to generate 2D patterns and 3D geometries for potential application as a template or porous host for nanomaterials.^[^ ^2^, ^3^, ^4^, ^5^ ^]^ By coupling molecules such as synthetic polymers, peptides and proteins, lipids, or saccharides to nucleic acids (**Figure** **1**), new functional materials can be created.

These hybrid materials, which combine various physicochemical properties and biological activities within a single unit, find a wide range of applications as biosensors,^[^ ^6^, ^7^, ^8^ ^]^ drug delivery systems,^[^ ^9^, ^10^, ^11^, ^12^, ^13^, ^14^ ^]^ self‐healing nanogels,^[^ ^15^, ^16^, ^17^ ^]^ and defined functional architectures such as micelles or patterned polymers.^[^ ^18^, ^19^ ^]^ For instance, polymer‐nucleic acid conjugates can form hydrogels and nanocarriers.^[^ ^9^, ^10^, ^14^, ^15^, ^16^ ^]^ Conjugates with (cationic) cell‐penetrating peptides (CPPs) enhance intracellular delivery,^[^ ^20^, ^21^, ^22^ ^]^ while therapeutic peptides, such as antigens, can induce immune responses when conjugated to DNA origami.^[^ ^23^ ^]^ Protein conjugates are used in creating enzyme cascades on DNA origami^[^ ^24^, ^25^ ^]^ and for antibody‐mediated targeted delivery of nucleic acids.^[^ ^26^ ^]^ Lipid and saccharide conjugates are primarily used to mediate interactions with cell membranes.^[^ ^27^, ^28^ ^]^

Nucleic acid conjugates include a wide variety of molecular architectures, which are determined by the type of nucleic acids, their intended functions, and the nature of the conjugated macro‐ or biomolecules.^[^ ^29^ ^]^ DNA, with its chemical stability, predictable base‐pairing hybridization and ease of modification, serves as a versatile scaffold for the self‐assembly of programmable nanostructures and for sensing applications.^[^ ^3^, ^8^, ^30^ ^]^ RNA, although more labile in biological fluids, offers unique structural and catalytic properties that are increasingly leveraged in therapeutics and RNA nanotechnology applications.^[^ ^31^, ^32^ ^]^ In terms of functional properties, antisense oligonucleotides (ASOs) and small interfering RNAs (siRNAs) are short, chemically modified DNA or RNA analogues designed to modulate gene expression by hybridizing with their target mRNA, thereby blocking its mRNA translation.^[^ ^33^, ^34^ ^]^ To enhance their therapeutic potential, these molecules (ASOs and siRNAs) are often conjugated to lipids,^[^ ^32^, ^35^ ^]^ polymers,^[^ ^36^ ^]^ or peptides.^[^ ^37^ ^]^ Such conjugation facilitates cellular uptake (i.e., penetration of the plasma membrane),^[^ ^37^ ^]^ enhances targeted delivery (e.g., into the cytoplasm), and improves stability under physiological conditions,^[^ ^36^ ^]^ while preserving their biological activities, including binding affinity and specificity.^[^ ^36^ ^]^ Messenger RNA (mRNA), on the other hand, has recently gained prominence as both a vaccine platform and a therapeutic agent for the prevention and treatment of a wide range of diseases.^[^ ^38^ ^]^ Lipid or polymer carriers enable the encapsulation of mRNA, thereby improving its stability (e.g., facilitating endosomal escape) and translational efficiency.^[^ ^38^ ^]^ However, the direct conjugation of mRNA to lipids or polymers remains relatively unexplored. Aptamers, analogous to antibodies, are single‐stranded DNA or RNA molecules that fold into unique tertiary conformations through intramolecular interactions.^[^ ^39^ ^]^ They have therefore been employed as functional nucleic acid scaffolds for the targeted delivery of conjugated polymer nanoparticles, enabling specific recognition of proteins or receptors on the cell membrane.^[^ ^39^ ^]^ The potency of nucleic acid therapeutics can be further optimized through chemical modifications of the oligonucleotide backbone, such as the incorporation of locked nucleic acids (LNAs) or phosphorothioate linkages, thereby expanding the design possibilities for functional conjugates.^[^ ^33^ ^]^

The synthesis of nucleic acid conjugates can be achieved through various approaches.^[^ ^40^, ^41^ ^]^ One common method involves synthesizing the building blocks separately and then coupling them in the final step,^[^ ^42^ ^]^ while another approach forms the respective blocks in situ.^[^ ^19^ ^]^ The coupling of defined building blocks requires specific orthogonal functional groups, which perform bioorthogonal reactions, such as click chemistry, to form the linkage.^[^ ^43^, ^44^, ^45^ ^]^ Although nucleic acids can be connected to other macromolecules via non‐covalent interactions, such as biotin‐streptavidin binding,^[^ ^46^ ^]^ covalent conjugation methods are more stable and customizable.^[^ ^47^ ^]^ The structural diversity of nucleic acids arises from variations in the backbone, the ribose sugar moiety, and the nucleobases.^[^ ^48^ ^]^ Such chemical modifications can enhance nuclease resistance but may also alter charge density, polarity, and hydrophobicity, thereby critically influencing conjugation reactivity, solubility, and purification strategies.^[^ ^48^, ^49^, ^50^ ^]^ Awareness of these nucleic acid‐specific effects is essential when adapting conjugation and purification protocols originally developed for unmodified DNA or RNA.

For most applications, the purification of nucleic acid conjugates from unreacted components or side products is essential, as impurities can significantly affect both experimental reliability and functional performance.^[^ ^51^ ^]^ Depending on the system, such impurities may alter responsiveness, interfere with molecular interactions, or compromise biocompatibility. In stimuli‐responsive systems such as PNIPAM conjugates, residual polymer can disrupt switching behavior.^[^ ^52^, ^53^ ^]^ In self‐assembling peptide‐nucleic acid systems, contaminants can prevent proper structure formation and impair functionality.^[^ ^54^ ^]^ In nanomedicine, incomplete purification may reduce therapeutic efficacy or lead to off‐target effects and toxicity.^[^ ^11^ ^]^ For example, in antibody‐mediated delivery, unconjugated oligonucleotides can accumulate in non‐target tissues, reducing specificity and increasing side effects.^[^ ^55^ ^]^ Excess CPPs, used to promote cellular uptake and endosomal escape, can be cytotoxic if not completely removed.^[^ ^56^ ^]^ Similarly, saccharide‐ and lipid‐conjugated oligonucleotides require high purity to ensure correct cell membrane recognition and interaction.^[^ ^27^, ^28^ ^]^ In nucleic acid nanotechnology, the removal of unconjugated strands is critical to achieve precise stoichiometry, structural uniformity, and hybridization fidelity. Across all these applications, purity directly impacts biosafety, therapeutic indices, and the reproducibility of results.^[^ ^57^ ^]^

Given the diverse properties of nucleic acid conjugates, purification presents several challenges, including undesired aggregation or precipitation, solubility issues, and difficulty separating products from excess reactants.^[^ ^58^, ^59^ ^]^ Common strategies exploit differences in size (hydrodynamic radius), charge, or polarity between the target conjugates and impurities.^[^ ^60^ ^]^ The appropriate method often depends on the physicochemical properties of the conjugate, as well as downstream application requirements. This review explores synthesis and purification strategies for nucleic acid conjugates with polymers, peptides, proteins, lipids, and saccharides. Previous reviews have comprehensively addressed the synthesis of bioconjugates^[^ ^61^, ^62^, ^63^ ^]^ and the functionalization of DNA origami structures.^[^ ^64^ ^]^ Our work complements these by focusing on the purification of nucleic acid bioconjugates, an aspect that has received comparatively limited attention and for which detailed experimental information is often lacking in published works. Since only the successful methods usually emerge in the literature, comprehensive information about why some other methods were not applied can often only be assumed. Here, we compile available methodologies and provide practical guidance for researchers.

## 2 Nucleic Acid Bioconjugate Synthesis and Purification

### 2.1 Synthesis Strategies

#### 2.1.1 General

To conjugate nucleic acids with functional molecules, well‐defined reaction conditions and approaches are essential for achieving high yields. Depending on the highly variable properties of the individual blocks within a conjugate, certain coupling methods are typically more effective than others. The amphiphilicity, size, accessibility, and overall architecture of the individual blocks play crucial roles. For instance, coupling highly hydrophobic molecules with hydrophilic DNA can lead to complications such as micelle formation.^[^ ^41^ ^]^ If the synthesis method is not optimally chosen, such incompatibilities may result in substantial yield losses or even complete reaction failure. Various strategies have been developed for nucleic acid functionalization, including solution‐phase and solid‐support synthesis, depending on the type, properties and desired modification site of the nucleic acid.^[^ ^13^, ^40^, ^41^, ^65^, ^66^, ^67^ ^]^ The three principal approaches, bioconjugation in solution, *grafting from*, and solid‐phase coupling, are outlined below and evaluated with respect to their strengths and practical applicability.

One of the most widely used strategies is direct bioconjugation in solution, often referred to as the *grafting to* approach in the context of polymers (Figure 1).^[^ ^43^, ^44^ ^]^ In this strategy, pre‐synthesized building blocks bearing accessible reactive end groups are coupled to form covalent bonds. Since both components can be fully characterized prior to conjugation, this method enables the preparation of well‐defined, controllable materials with tailored properties. Common coupling reactions include *N‐*hydroxysuccinimide (NHS)‐amine, thiol‐maleimide, and alkyne‐azide click chemistries.^[^ ^44^ ^]^ As the accessibility and reactivity of the functional groups are crucial, careful optimization of reaction conditions and solvents is typically required to avoid side reactions or low yields caused by steric hindrance or limited accessibility. Thiol‐maleimide coupling, for instance, can be challenging for coupling nucleic acids with peptides or proteins, since cysteine side chains may promote undesired side reactions or multiple couplings.^[^ ^68^ ^]^ Similarly, dibenzocyclooctyne (DBCO) groups used for click coupling can exhibit π‐π‐stacking or reduced accessibility in solution due to their hydrophobic aromatic character. Flexible linkers are often introduced to enhance reaction efficiency by improving the spatial accessibility of functional groups. The length and chemical nature of the linker not only influence coupling yields but can also modulate the structural properties of the resulting conjugate, such as rigidity or flexibility. The chemical versatility and adaptability to diverse block properties represent major advantages of solution‐phase coupling. Furthermore, this approach is well‐suited for scale‐up, as it allows for proportional adjustment of reactant concentrations relative to solvent volume, making it a robust and adaptable method for larger‐scale synthesis. However, since an excess of the more available building block is used to achieve high yields, purification can become challenging due to substantial amounts of unreacted residues in solution.^[^ ^42^, ^69^, ^70^ ^]^ It is therefore important to consider which component should be used in excess with regard to the subsequent separation of unreacted molecules, especially when both are available in larger quantities. For instance, solubility can play a decisive role in enabling precipitation or efficient purification using automated systems.

To address some of the challenges mentioned above, polymer‐nucleic acid conjugates, for instance, can be prepared using the *grafting from* method.^[^ ^19^ ^]^ In this strategy, the nucleic acid is first covalently modified with a polymerization agent, such as a chain transfer agent (CTA), allowing polymer chains to grow directly from the nucleic acid upon addition of monomers in solution. This in situ polymerization often yields high reaction efficiency, and the main by‐products are unreacted monomers, which can typically be removed by ultrafiltration.^[^ ^19^, ^71^ ^]^ Since polymerization, often radical polymerization, occurs directly from the nucleic acid, this method is highly sensitive to impurities and oxygen. Oxygen removal can be achieved by freeze‐thaw pump cycles or enzyme‐based degassing systems.^[^ ^72^ ^]^ Maintaining uniform reaction conditions on a larger scale can be challenging but is essential for obtaining well‐defined conjugates. Issues such as uneven temperature distribution or impurities may increase side reactions and reduce overall product consistency. Moreover, the activated nucleic acid block usually needs to be synthesized and purified separately under mild conditions to preserve molecular integrity. The high purity requirements and additional preparation steps make upscaling difficult and demand skilled handling of all components and reaction procedures. The *grafting from* approach further offers only limited control over polymer chain length and dispersity, which can be critical for applications requiring uniformity. When precise control over polymer architecture is required, alternative strategies such as *grafting to* or solid‐supported synthesis are preferred.^[^ ^41^ ^]^

Peptide‐ or protein‐nucleic acid conjugates are typically formed by coupling the nucleic acid to the biomolecule via reactive end groups, either in solution^[^ ^73^ ^]^ or on a solid support.^[^ ^42^ ^]^ One common solid‐supported approach involves conjugating the peptide or protein directly to an immobilized nucleic acid strand.^[^ ^74^ ^]^ Since oligonucleotides are generally prepared by automated solid‐phase phosphoramidite chemistry, where iterative cycles of coupling, capping, and oxidation yield defined sequences, this platform is well suited for subsequent conjugate formation.^[^ ^75^ ^]^ The DNA block can thus be reacted with the desired molecular partner immediately after synthesis. Understanding this process provides important context for post‐synthetic conjugation strategies and purification choices, as the protecting groups and chemical modifications introduced during synthesis strongly affect reactivity and solubility. When specific sequences are required, for example to introduce defined peptide motifs at the nucleic acid terminus, phosphoramidite‐modified monomers can be used.^[^ ^65^, ^76^ ^]^ These monomers enable the stepwise incorporation of functional blocks during solid‐phase synthesis, allowing for the construction of complex architectures such as multivalent or brush‐like conjugates. The advantage of solid‐phase synthesis lies in its built‐in purification through iterative washing steps after each coupling cycle. This minimizes the accumulation of unreacted reagents and simplifies downstream purification after final cleavage from the support. Moreover, solid‐phase methods offer scalability: if enough reactive monomers are available, larger batches can typically be synthesized without compromising coupling efficiency or product quality. Beyond peptides and proteins, this approach is also well‐suited for the conjugation of nucleic acids with other functional groups—including lipids,^[^ ^13^, ^77^ ^]^ saccharides^[^ ^28^, ^78^ ^]^ and synthetically demanding polymers.^[^ ^79^ ^]^ Its versatility and compatibility with diverse building blocks make solid‐phase synthesis a powerful tool for the generation of structurally defined nucleic acid conjugates across a wide range of applications.

The synthesis of non‐covalent ionic nucleic acid complexes relies on electrostatic interactions between the negatively charged phosphate backbone of DNA or RNA and positively charged ligands or carriers, without forming a covalent chemical bond.^[^ ^80^, ^81^, ^82^ ^]^ Typically, the nucleic acid is placed in a suitable buffer, followed by addition of a cation‐rich partner (e.g., poly‐l‐lysine, poly‐arginine, or a cationic polymer or nanoparticle surface) to form complexes through electrostatic association. Complexation may be further stabilized by complementary oligonucleotides or ionic structures. Several parameters critically influence complex formation, including the charge density of the cationic component, the ionic strength, and the pH of the buffer (as these influence the electrostatic binding). This strategy offers considerable flexibility and modularity but requires careful optimization of binding conditions, and the resulting complexes are sensitive to environmental changes and may dissociate under unfavorable conditions.

#### 2.1.2 Practical Guidance for the Synthesis of Nucleic Acid Conjugates

The following examples illustrate general strategies for the synthesis of nucleic acid conjugates under different physicochemical and material constraints. These cases are intentionally simplified to demonstrate the underlying design principles and to guide the choice of synthesis strategy. Naturally, each conjugate system presents additional parameters and limitations that must be optimized individually. Nevertheless, this overview is intended to help readers develop an intuitive understanding of how molecular properties influence the synthetic approach.

*Target Conjugate A*

Polymer block: Hydrophilic, large (≈50 kDa), readily available

DNA block: 20 kDa, available

Desired amount: > 20 mg

Recommended approach: Since both components are hydrophilic, they remain soluble in aqueous media and the conjugate is not expected to display strong amphiphilic behavior such as micelle formation. Solution‐phase coupling is therefore the most suitable method. This approach is also well suited for scale‐up, allowing preparation of larger quantities (>20 mg). To increase yield, an excess of one component may be used. If the polymer contains sensitive or reactive side chains, reaction conditions should be carefully optimized to minimize side reactions or unintended couplings.

*Target Conjugate B*

Polymer block: Hydrophilic, small (≈5 kDa), very limited availability

Nucleic acid block: 20 kDa, available

Desired amount: ≈ 2 mg

Recommended approach: Both solution‐phase coupling and the *grafting from* strategy are feasible. The *grafting from* approach, however, requires a suitable monomer and prior functionalization of the nucleic acid with a polymerizable group, which can introduce additional synthetic and purification steps. Given the limited polymer availability, *grafting from* may still be advantageous, as it can achieve high efficiency and allows straightforward purification by spin filtration to remove unreacted monomers. Conversely, coupling in solution is simpler and may provide higher yields if the nucleic acid block is used in excess. The final choice depends on monomer availability, equipment, and purification preference.

*Target Conjugate C*

Polymer block: Hydrophobic, large (≈20 kDa), available

Nucleic acid block: 20 kDa, available

Desired amount: ≈ 2 mg

Recommended approach: The high hydrophobicity of the polymer will likely impart amphiphilic character to the conjugate, promoting micelle or aggregate formation. Direct coupling in aqueous solution is therefore not practical. In such cases, solid‐supported synthesis is the preferred method, as it permits reactions in organic solvents that dissolve the hydrophobic polymer and enable efficient coupling to the DNA block. This strategy also facilitates purification by allowing removal of excess reagents before cleavage from the support.

*Target Conjugate D*

Peptide block: Defined sequence, small (≈6 amino acids (AAs))

Nucleic acid block: 10 kDa

Desired amount: ≈2 mg

Recommended approach: Two strategies are generally suitable: coupling in solution or solid‐supported synthesis. Solution‐phase coupling is appropriate if the peptide is sufficiently hydrophilic; however, the presence of hydrophobic AAs can lead to solubility issues that hinder conjugation. Cysteine residues also require attention as thiol‐maleimide coupling should be avoided if unwanted side reactions or multiple linkages are likely. Solid‐supported synthesis, in contrast, allows stepwise addition of AAs in a defined sequence and can be carried out in organic solvents, which is advantageous for hydrophobic peptides.

*Target Conjugate E*

Protein: Surface‐modified, ≈80 kDa

Nucleic acid block: 10 kDa

Desired amount: ≈ 2 mg

Recommended approach: Proteins and nucleic acids generally exhibit good mutual solubility, allowing coupling reactions to proceed in solution. This approach also enables the nucleic acid to be used in excess to achieve higher degrees of surface modification. When selecting coupling chemistry, cysteine residues should be considered carefully; strain‐promoted azide‐alkyne cycloaddition (SPAAC) is typically preferred to preserve protein integrity. To maintain native folding, solvent composition and reaction conditions must be chosen to minimize denaturation or loss of biological activity.

These examples highlight how the physicochemical properties of each block, such as hydrophilicity, hydrophobicity, and molecular size, determine the optimal synthesis strategy. Selecting an appropriate method not only maximizes yield and structural definition but also simplifies purification and scalability.

### 2.2 Purification Strategies

#### 2.2.1 General

**Figure 2:**
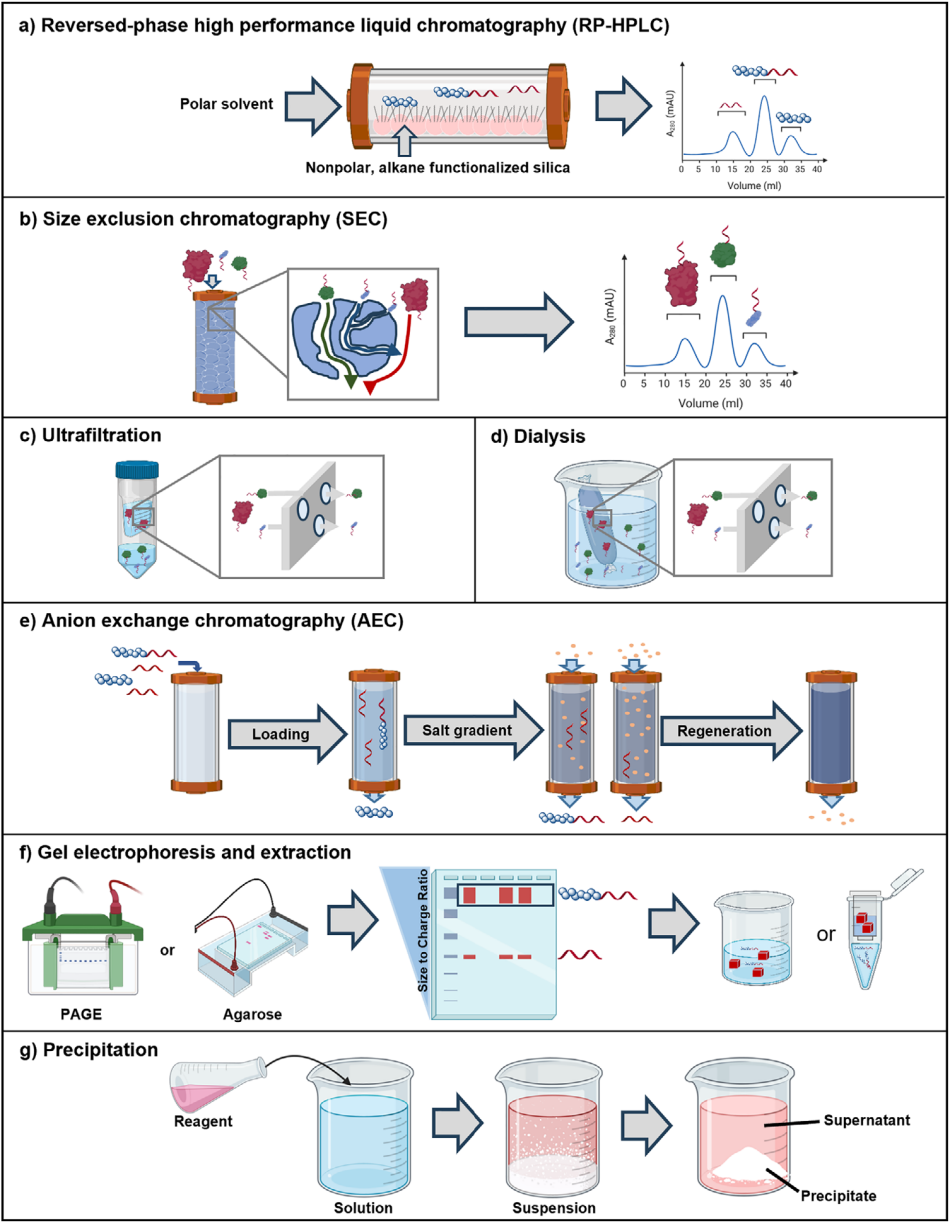
Schematic representation of purification principles. a) Reversed‐phase high‐performance liquid chromatography (RP‐HPLC), b) size exclusion chromatography (SEC), c) ultrafiltration (spin filtration), d) dialysis, e) anion exchange chromatography (AEC), f) gel electrophoresis and extraction, and g) precipitation. (Figure created with BioRender.com).

The choice of synthesis strategy for a given nucleic acid conjugate also influences which purification method is most suitable. Various purification techniques are available, primarily exploiting differences in size (hydrodynamic radius), charge, or hydrophobicity between the target conjugate and residual impurities. Commonly used methods include reversed‐phase high‐performance liquid chromatography (RP‐HPLC),^[^ ^83^, ^84^ ^]^ size exclusion chromatography (SEC),^[^ ^85^ ^]^ ultrafiltration,^[^ ^69^, ^86^ ^]^ dialysis,^[^ ^87^ ^]^ anion exchange chromatography (AEC),^[^ ^88^, ^89^ ^]^ gel electrophoresis,^[^ ^90^, ^91^ ^]^ and precipitation.^[^ ^92^, ^93^, ^94^ ^]^ Each method offers distinct advantages and limitations in terms of resolution, scalability, and compatibility with different conjugate types. A comparative overview of these techniques, their key characteristics, and suitability for various nucleic acid conjugates are provided in **Figure** **2** and **Tables** **1** and **2**. It should be noted that Table 2 does not differentiate between nucleic acid lengths or types. However, longer or structured nucleic acids, such as aptamers, often form complex secondary structures that significantly influence purification efficiency and require optimization of parameters.

**Table 1:** Purification strategies for nucleic acid conjugates. Overview of the main characteristics, advantages, and limitations for purifying nucleic acid conjugates. (Icons from BioRender.com).

	RP‐HPLC 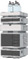	SEC 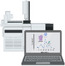	Ultrafiltration/ dialysis 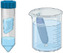	AEC 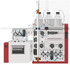	Gel electrophoresis and extraction 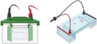	Precipitation 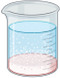
**Characteristics**						
Separation principle	differences in polarity	differences in hydrodynamic radius	differences in hydrodynamic radius	differences in charge	differences in charge and size	solubility
Optimization parameters	eluent gradient	pore size and loading of sample	pore size and loading of sample	eluent gradient and resin charge strength	gel material, concentrations, buffer system, voltage, and time used	solvent, pH, concentration
Upscaling	possible by changing size of the column; resolution may change	possible by changing size of the column; resolution may change	possible if membrane surface is adjusted	possible by changing the size of the column	difficult due to limited loading amount and gel size	possible
**Advantages**	▪ flexibility in column choice (C4, C8, C18) ▪ flexibility in choice of column temperature, buffer pH, and use of ion pair agents	▪ easy to use ▪ method requires little optimization ▪ architectures remain intact	▪ easy to use ▪ architectures remain intact ▪ can be used for solvent exchange	▪ easy separation of charged and uncharged components ▪ flexibility in choice ofcolumn and eluent type/saltconcentration	▪ challenging conjugates possible ▪ gel composition can be easily modified ▪ architectures remain intact	▪ very easy to use ▪ small and no special equipment required
**Limitations**	▪ chemically equal reactants & conjugates causing peak overlap ▪ excess of reactant can result in loss of separability of product ▪ method requires optimization	▪ excess of reactants can cause separation issues ▪ phase separating components can cause purification issues	▪ morphology‐forming conjugates not separated ▪ small loss of conjugate in membranes	▪ components need to be water‐soluble ▪ method needs optimization for getting best results	▪ small amounts ▪ time‐consuming	▪ co‐precipitation ▪ usually only for rough purification or preparation for next step

**Table 2:** Suitability of purification strategies for nucleic acid conjugates. Suitability recommendations are made independently of synthesis approaches (e.g., excess of reagents); detailed discussions are provided in the specific sections of this review. Legend: +++ highly suitable, ++ suitable, + suitable in some cases, ‐ unsuitable, N/A no information available. (Icons from BioRender.com).

		RP‐HPLC 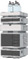	SEC 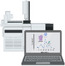		Ultrafiltration/ dialysis 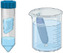	AEC 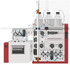		Gel electrophoresis and extraction 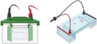	Precipitation 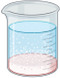
X‐DNA‐conjugates									
Polymer 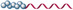	uncharged	+++	++	−			++	++	++
	polar	++	+++	++			+++	+++	+
	charged	−	N/A	N/A			++	++	+
Polymer 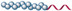	uncharged	+++	++	−			+	++	++
	polar	++	+++	++			++	+++	+
	charged	−	N/A	+			++	++	+
Peptide 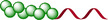		+++	++	++			++	++	+
Protein 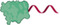		+	+++	+++			+++	++	N/A
Lipid 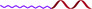		+++	N/A	−			N/A	++	N/A
Saccharide 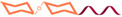		+++	N/A	N/A			++	++	+

#### 2.2.2 RP‐HPLC

One of the most widely used techniques for separating nucleic acid conjugates is RP‐HPLC and its ion‐pairing variant (IP‐RP‐HPLC) (Figure 2a).^[^ ^84^, ^95^ ^]^ In RP‐HPLC, separation occurs on a hydrophobic stationary phase where molecules are primarily separated according to their hydrophobicity. However, since nucleic acids are highly hydrophilic due to their negatively charged phosphate backbone, they show only limited retention under standard RP conditions.^[^ ^83^ ^]^ In certain cases, this can be advantageous when the building blocks are of comparable size, as the conjugate, which combines the chemical properties of both components, elutes distinctly from the unreacted species, allowing for chromatographic separation and purification. In contrast, when the building blocks differ significantly in size or when the nucleic acid component is relatively small, both conjugate and the larger component may show similar retention times, complicating separation.^[^ ^88^ ^]^

To overcome this limitation, cationic ion‐pairing reagents such as triethylammonium acetate (TEAA), triethylamine hexafluoroisopropanol (TEA/HFIP), or hexylammonium acetate (HAA) are commonly employed in IP‐RP‐HPLC. These reagents form ion pairs in solution with the negatively charged phosphate groups of nucleic acids, thereby increasing their overall hydrophobicity. As a result, the nucleic acid interacts more strongly with the hydrophobic stationary phase, enabling more effective retention and improved separation of the conjugated and unconjugated species.^[^ ^96^, ^97^ ^]^

In practice, columns with different stationary‐phase functionalization can be employed. The most common are C18 (octadecylsilane), C8 (octylsilane), and C4 (butylsilane) columns. C18 phases are highly hydrophobic and interact strongly with the hydrophobic segments of a conjugate, whereas C8 and C4 columns provide weaker interactions. Consequently, C8 or C4 columns are often preferred for larger or more hydrophobic conjugates, where C18 phases may retain the analytes too strongly.^[^ ^98^, ^99^, ^100^, ^101^ ^]^ The mobile phase typically consists of a buffered aqueous system, either slightly acidic or basic, combined with an organic solvent such as acetonitrile (ACN). Purification is generally performed using a linear ACN gradient, with more hydrophobic conjugates eluting later than unmodified nucleic acids. UV detection at 260 nm enables accurate monitoring of nucleic acid absorbance, while conjugates containing peptide or aromatic components can be detected at 214–220 nm. In addition, mass‐spectrometric detection (LC‐MS) can be applied, provided that volatile ion‐pair reagents such as TEA/HFIP are used, as non‐volatile salts interfere with ionization. After fraction collection, the desired samples are typically concentrated by ultrafiltration, followed by lyophilization or vacuum drying to remove ion‐pair reagents and residual buffer salts.^[^ ^88^ ^]^

The main advantages of (IP‐)RP‐HPLC are its high resolution, short run times, and excellent reproducibility, which enable detection of even subtle differences in the hydrophobicity profiles of modified oligonucleotides. However, method development is often required to optimize buffer composition and gradient conditions, typically involving multiple test runs with small sample amounts to achieve optimal peak separation. During scale‐up, separation efficiency and peak sharpness commonly decrease with higher sample loads, which can limit the applicability of this technique for mixtures containing a large excess of one component.^[^ ^88^ ^]^ In such cases, careful adjustment of flow rate and column dimensions is essential. It should also be noted that nucleic acids may exhibit heterogeneous retention due to secondary structures, such as G‐quadruplexes. Increasing the column temperature, often up to 60 °C, can help minimize such structural effects and improve peak resolution.^[^ ^97^, ^102^ ^]^

In summary, RP‐HPLC and its ion‐pairing variant (IP‐RP‐HPLC) represent powerful, versatile, and widely used methods for the purification of nucleic acid conjugates. While conventional RP‐HPLC is suitable only in selected cases, the incorporation of ion‐pair reagents enables efficient chromatographic separation and significantly broadens the applicability of this technique.

#### 2.2.3 SEC

The separation principle of SEC (Figure 2b) is based on the hydrodynamic size of the analytes: larger molecules penetrate the pores of the stationary phase only partially and therefore elute earlier, whereas smaller molecules diffuse deeper into the pores and elute later. This property makes SEC particularly suitable for removing small by‐products such as salts, unreacted reagents, or low molecular weight (MW) impurities, as well as for separating aggregates or multimers from monomeric conjugates. Since the separation is purely governed by size rather than chemical interactions, the native structure and overall architecture of the conjugate are largely preserved ‐ often a significant advantage over other chromatographic methods.^[^ ^103^ ^]^

Various column materials are available for the purification of nucleic acid conjugates, with pore size and chemical composition being key determinants of separation performance. Classic SEC media such as Sephadex, Sephacryl, and Superdex are based on cross‐linked dextran or agarose polymers and are well‐suited for preparative purification and desalting steps.^[^ ^103^ ^]^ These materials are available in a range of pore sizes, making proper selection critical for achieving adequate resolution.^[^ ^85^, ^104^, ^105^ ^]^ If the pore size is too small, the target molecules elute too early in the dead volume; conversely, pores that are too large lead to insufficient separation, as all components pass through the column at the same time. Columns with small pores (≈130 Å) are optimal for small conjugates (<8 kDa) or for separating low MW reactants, whereas 300 Å pores are typically used for medium‐sized conjugates. Very large pores (500–1000 Å) are recommend for larger complexes, such as protein‐nucleic acid conjugates or nanoparticles.

Equally important as the stationary phase is the composition of the mobile phase. For nucleic acid conjugates, aqueous buffers of low to moderate ionic strength are typically used, with NaCl additives helping to stabilize hydrophobic conjugates. In some cases, elevated column temperatures (40–60 °C) are used to reduce secondary structures of the nucleic acid, thereby improving resolution.^[^ ^85^, ^104^, ^106^ ^]^

Overall, SEC provides a versatile and gentle purification platform for nucleic acid conjugates, particularly effective for removing small by‐products and salts or when maintaining the native architecture of the conjugate is essential.^[^ ^107^ ^]^ However, the presence of a large excess of one reactant can lead to broadened or less distinct peaks, complicating efficient separation.^[^ ^88^ ^]^

#### 2.2.4 Ultrafiltration and Dialysis

Two straightforward and gentle methods for purifying nucleic acid conjugates or exchanging buffers are ultrafiltration and dialysis. Both rely on the selective permeation of small molecules through semipermeable membranes, allowing efficient removal of low MW impurities while preserving the structural integrity of the conjugates. Membrane pore size and solute diffusion are determined by the hydrodynamic radius of the solute. MW serves as an approximate descriptor, valid primarily for molecules of similar shape, density and hydration.

Ultrafiltration (spin filtration) (Figure 2c) uses semi‐permeable membranes with defined MW cutoffs (MWCO) and applies pressure, typically via centrifugal force or tangential flow, to drive solvents and small components such as salts or unreacted reagents through the membrane, while larger molecules, including the desired conjugate, are retained.^[^ ^86^ ^]^ The key advantages of ultrafiltration are its speed, flexibility, and ease of use. Commercial centrifugal filters (e.g., Amicon Ultra, Vivaspin) enable rapid concentration and desalting of samples in the micro‐ to milliliter range. A membrane with an MWCO significantly lower than the conjugate's molecular size is chosen to minimize sample loss. Extensive buffer exchange can be achieved through repeated filtration steps (diafiltration), in which fresh buffer is added between cycles. Limitations arise when the conjugate and residual substances differ greatly in size, as very large conjugates may lead to slow flow rates or membrane clogging. In addition, concentration gradients and fouling (accumulation of components on the membrane surface) often occur, reducing the effective flow.^[^ ^69^, ^88^ ^]^ For larger volumes or to avoid these effects, tangential flow filtration (TFF) is used, in which the fluid flows tangentially across the membrane, minimizing surface deposits.^[^ ^108^ ^]^

Dialysis (Figure 2d) operates on a similar principle. The conjugate is placed in a dialysis tube or bag with a semipermeable membrane. This is immersed in a large volume of solvent, usually buffer. Small molecules diffuse outward along their concentration gradient, while the larger conjugate remains within the dialysis chamber.^[^ ^109^ ^]^ Replacing the external solvent several times enhances the removal of small impurities and allows effective buffer exchange. Dialysis is particularly valuable for gentle purification and buffer adjustment following chemical modification or chromatographic separation.

In practice, ultrafiltration and dialysis are often combined or used as complementary pre‐ or post‐chromatographic steps. Both techniques provide simple and cost‐effective options for purification and buffer exchange of nucleic acid conjugates. Their performance depends critically on the appropriate choice of membrane MWCO, sample‐to‐buffer ratio, and concentration conditions. Similar to SEC, these membrane‐based methods maintain the native architecture and biological functionality of nanoscale architectures and macromolecules.^[^ ^110^, ^111^ ^]^

#### 2.2.5 AEC

AEC is one of the key methods for purifying nucleic acids and their conjugates (Figure 2e). It relies on electrostatic interactions between the negatively charged phosphate backbone of nucleic acids and positively charged groups on the stationary phase. Typically, strongly basic ion exchangers containing quaternary ammonium groups on silica or polymer‐based carrier materials are used. These materials are commonly Q‐ or DEAE‐functionalized (Q = quaternary ammonium; DEAE = diethylaminoethyl) and offer high binding capacities.^[^ ^112^ ^]^

In AEC, conjugates are first loaded onto the column in a buffer of low ionic strength, allowing binding to the positively charged groups. Uncharged reactants are removed by washing, while elution is achieved by gradually increasing the salt concentration or pH, which weakens the electrostatic bonds. The more hydrophobic, generally uncharged block of the nucleic acid conjugate influences the retention time, thereby facilitating the separation of unmodified oligonucleotides and reaction by‐products from the conjugate.^[^ ^88^ ^]^ Common eluents include buffers such as Tris (tris(hydroxymethyl)aminomethane), BIS‐TRIS‐propane (1,3‐bis[tris(hydroxymethyl)methylamino]propane), or HEPES (N‐(2‐hydroxyethyl)‐piperazine‐N′‐(2‐ethanesulfonic acid)), combined with sodium chloride or sodium perchlorate as counterions. The choice and concentration of the salt determine the separation efficiency and elution profile.^[^ ^113^, ^114^ ^]^

AEC provides high selectivity and is readily scalable, from analytical scale to preparative purification, making it a powerful and versatile technique for separating and purifying nucleic acid conjugates. It is particularly effective for removing unbound reactants and by‐products, especially more heavily charged or longer oligonucleotides.^[^ ^79^, ^115^, ^116^ ^]^ However, it should be noted that higher‐order DNA structures, such as DNA origami, may lose their architecture due to interactions with the column and gradients of salt concentrations.

#### 2.2.6 Gel Electrophoresis

Gel electrophoresis separates nucleic acids based on their migration through a porous gel matrix under an electric field, where negatively charged molecules move toward the anode (Figure 2f).^[^ ^117^, ^118^ ^]^ Molecules with a smaller size‐to‐charge ratio migrate faster and farther than larger ones. This technique can be used to purify nucleic acid conjugates by separating the desired product from unreacted oligonucleotides. Uncharged molecules, such as most polymers, do not migrate and remain at their initial position. After electrophoresis, the desired bands are visualized under UV light or by visible staining, excised, and the target fragment is recovered by gel extraction or electroelution. Two main types of gels are commonly used: agarose and polyacrylamide.

Agarose gel electrophoresis is particularly suitable for larger nucleic acid fragments (≈100 bp to several kilobases) due to its relatively coarse pore structure. It is often used for the preparative separation and purification of larger conjugates or DNA fragments. The agarose concentration (typically 0.7–2%) determines the pore size and thus the resolution. The use of “low‐melting” agarose is particularly useful, as it can be liquefied by gentle heating after electrophoresis, facilitating recovery of the conjugate. The extracted fragment is usually purified using a silica membrane (e.g., via commercial gel extraction kits) to remove residual agarose.^[^ ^90^, ^91^, ^119^ ^]^

In contrast, polyacrylamide gel electrophoresis (PAGE) provides a much finer pore structure, allowing high‐resolution separation of small nucleic acids (typically <100 bp) and their conjugates. It is therefore preferred when only small differences in length or mass need to be resolved. Extraction from polyacrylamide gels is more challenging because the gel is not fusible. Common recovery methods include the “crush‐and‐soak” approach, in which the gel fragment is crushed and incubated in buffer to release the conjugate, or electroelution, where an electric field transfers the nucleic acid from the gel matrix into a collection buffer.^[^ ^119^, ^120^ ^]^

The choice between agarose and PAGE depends on the size and characteristics of the conjugate: agarose gels are ideal for larger and less sensitive molecules, whereas PAGE offers superior resolution for short oligonucleotides or subtle structural variations.^[^ ^69^, ^71^ ^]^ To achieve optimal separation, gel concentration, running buffer, and applied voltage should be carefully adjusted. Overall, gel electrophoresis, particularly when combined with targeted gel extraction, represents a versatile, cost‐effective, and reliable method for purifying nucleic acid conjugates. However, due to loading limitations, it is generally restricted to small sample quantities; overloading the gel leads to diffuse bands and reduced recovery efficiency.

#### 2.2.7 Gravity Based Methods

Gravity‐based chromatography columns represent a straightforward approach for purifying nucleic acid conjugates on both analytical and preparative scales. In contrast to high‐pressure methods such as HPLC, the sample solution flows solely by gravity, eliminating the need for pumps or complex instrumentation.^[^ ^121^, ^122^ ^]^ The setup typically consists of a pre‐packed or self‐filled column with filter frits containing a defined chromatography resin. The columns are easy to handle and require no specialized equipment. Before sample loading, the column is equilibrated with buffer to activate the resin and remove storage components such as ethanol. The sample solution should be free of particulates to prevent clogging, and elution is performed manually by adding buffer with adjusted salt or pH conditions.

Depending on the resin's chemical functionality, different separation mechanisms can be used, most commonly AEC and hydrophobic interaction chromatography (HIC). In AEC, negatively charged nucleic acids bind to positively charged functional groups on the stationary phase, as described above. The sample is applied in a low ionic strength buffer to promote binding, and bound molecules are eluted by gradually increasing the salt concentration or adjusting the pH.^[^ ^123^, ^124^, ^125^ ^]^

HIC, in contrast, relies on the reversible interaction of molecules with hydrophobic ligands on the resin in the presence of high salt concentrations. The elevated salt reduces the hydration shell around the molecules, enhancing hydrophobic interactions between the stationary phase and hydrophobic regions of the analyte. Subsequent reduction of the ionic strength or the addition of mild organic modifiers facilitates elution. While unmodified nucleic acids exhibit weak binding to HIC resins due to their high hydrophilicity, this method is particularly suitable for purifying nucleic acid conjugates bearing hydrophobic modifications such as peptides, lipids, or synthetic polymers.^[^ ^126^, ^127^ ^]^

The advantages of gravity‐based chromatography include simplicity, low cost, and broad applicability for small to medium volumes. However, limitations include minimal flow control, lower resolution compared to high‐pressure systems, and the potential for channeling or uneven flow if the resin packing is suboptimal. Furthermore, fine‐tuning of elution gradients is restricted relative to pumped systems. Despite these constraints, gravity‐based chromatography provides a practical, cost‐effective, and flexible alternative for the purification of nucleic acid conjugates, particularly in laboratories without access to automated instrumentation.

#### 2.2.8 Precipitation Based Methods

Precipitation‐based purification relies on reducing the solubility of the nucleic acids by disrupting their hydration shell, which promotes aggregation and subsequent precipitation. Typically, an organic solvent such as ethanol, isopropanol, or methanol is added in combination with salts (e.g., sodium or ammonium salts). Under these conditions, larger nucleic acids, the conjugates, and DNA architectures aggregate and precipitate, allowing their collection by centrifugation, while smaller nucleic acids remain in the supernatant.^[^ ^92^, ^93^, ^94^ ^]^

Alternatively, precipitation can be induced using polyethylene glycol (PEG), which reduces nucleic acid solubility through volume exclusion effects. In combination with salts such as NaCl, PEG precipitation can be used selectively to isolate larger nucleic acids or assembled architectures, while smaller fragments remain soluble.^[^ ^69^, ^93^, ^110^ ^]^

Precipitation methods offer several advantages: they are rapid, simple, and require no specialized equipment. However, they also present limitations, including product losses at low concentrations, potential co‐precipitation of impurities or unreacted components, and difficulties in redissolving the pellet. When several chemically similar species are present, selective separation becomes particularly challenging. For optimal yield, parameters such as temperature, salt concentration, and the choice of precipitating agent should be carefully optimized.

#### 2.2.9 Selection of Purification Method

The selection of an appropriate purification method for nucleic acid conjugates depends on numerous factors and must typically be tailored to the specific system. Because such conjugates can differ widely in size, charge, hydrophobicity, and chemical stability, no single universal method exists. Instead, several complementary approaches, such as RP‐HPLC, AEC, SEC, ultrafiltration, gel electrophoresis, or precipitation, are available, each offering distinct advantages and limitations depending on the molecular characteristics and purification goals. In this review, we present these methods, outlining their underlying separation principles and comparing their respective strengths and weaknesses.

In practice, the choice of purification strategy often depends on available laboratory resources and instrumentation. Chromatographic techniques such as RP‐HPLC and AEC provide high resolution and reproducibility but require specialized equipment and technical expertise. Simpler methods, including ethanol or PEG precipitation, dialysis, or ultrafiltration, can be performed rapidly and without complex infrastructure, although they typically offer lower selectivity.

Because purification efficiency and selectivity strongly depend on the physicochemical properties of a given conjugate, it is often necessary to experimentally compare and optimize multiple methods when working with new systems. In many cases, a combination of techniques, for example, precipitation for pre‐purification followed by chromatographic separation, proves most effective for achieving high purity and yield. For conjugates that have already been reported, the existing literature can provide valuable guidance in selecting suitable purification methods. Even for chemically related systems, established protocols can often be applied with only minor adjustments. In the following sections, we therefore present a range of representative examples for different classes of nucleic acid conjugates, providing a practical reference for identifying and comparing purification strategies.

## 3 Nucleic Acid Bioconjugate Classes

### 3.1 Polymer‐Nucleic Acid Conjugates

#### 3.1.1 General

Polymer‐nucleic acid conjugates have gained significant attention for their potential applications as biosensors,^[^ ^8^ ^]^ hydrogels,^[^ ^128^ ^]^ and nanocarriers.^[^ ^129^, ^130^ ^]^ For instance, by combining highly hydrophilic nucleic acid blocks with orthogonal hydrophobic polymer blocks, new hybrid amphiphilic materials capable of forming micelles,^[^ ^131^ ^]^ vesicles,^[^ ^8^ ^]^ or stimuli‐responsive gels^[^ ^53^ ^]^ can be created, while ensuring a defined size, dispersity and thermo‐responsiveness. To fabricate polymer‐nucleic acid conjugates, various synthetic strategies exist. The *grafting from* method uses single‐stranded nucleic acids equipped with a functional molecule like a CTA to perform reversible addition‐fragmentation chain‐transfer (RAFT) polymerization in solution. This method requires both the DNA‐CTA complex and the monomers to be soluble in protic solvents such as water or dimethylformamide (DMF).^[^ ^71^ ^]^ *Grafting from* is also applied to controlled radical polymerization strategies such as atom transfer radical polymerization (ATRP).^[^ ^132^ ^]^ Due to the small starting materials, purification is usually very easy to carry out using ultracentrifugation. Alternatively, the *grafting to* strategy uses nucleic acid and polymer building blocks with reactive end groups for conjugation.^[^ ^69^ ^]^

Another approach, primarily used for amphiphilic conjugates, involves solid‐supported couplings with the nucleic acid building block immobilized on a controlled pore glass (CPG) bead, as used in DNA/RNA synthesis, while the polymer block is coupled to the reactive end.^[^ ^41^ ^]^ Conjugation can be performed in organic solvents, enhancing the yield for lipophilic polymers. The final washing step simplifies purification by removing impurities from the coupling.^[^ ^74^ ^]^ Another CPG‐supported strategy involves creating phosphoramidite‐functionalized monomers and coupling these to the solid‐supported nucleic acids step‐by‐step, building a defined sequence of polymeric and nucleic acid units.^[^ ^133^ ^]^ This technique is crucial for incorporating demanding monomers where conjugate synthesis would otherwise be challenging. Given the various synthesis methods and resulting conjugate properties, purification strategies vary. Relevant purification strategies for nucleic acid conjugates with polymers, ranging from lipophilic to hydrophilic and charged, are outlined below.

#### 3.1.2 Hydrophobic Polymers

Conjugating hydrophobic polymers to nucleic acids yields a class of hybrid materials with unique features such as phase separation and stimuli responsiveness.^[^ ^134^ ^]^ The emerging amphiphilicity enables modulation of nanoscale morphology by changes in the nucleic acid block from single‐stranded to double‐stranded, as well as by varying the polymer length. The ability of these materials to form micelles facilitates the transport of lipophilic drugs or biomarkers, making them promising candidates as nanocarriers.^[^ ^134^, ^135^, ^136^ ^]^

However, the amphiphilic nature of such conjugates also presents specific challenges for purification. The balance between hydrophilic and hydrophobic domains critically affects solubility and interactions with chromatographic matrices. This can be partially mitigated during synthesis by employing solid‐support strategies, which allow unreacted components to be washed away in situ. For other synthesis routes, chromatographic methods such RP‐HPLC and SEC are frequently used, as they enable separation based on hydrophobicity or molecular size, respectively. Amphiphilic conjugates tend to exhibit stronger interactions with hydrophobic stationary phases, leading to broader or delayed elution peaks in RP‐HPLC; therefore, optimization of solvent composition and gradient strength is essential for efficient recovery.

Ultrafiltration and dialysis should be applied with caution, as the self‐assembly or micellar formation of amphiphilic conjugates with hydrophobic starting materials can hinder or prevent purification. For highly hydrophobic conjugates, precipitation‐based approaches (e.g., ethanol or PEG‐induced precipitation) may be employed, although recovery efficiency strongly depends on polymer composition and ionic strength. Overall, the purification strategy must be tailored to the conjugate's physicochemical properties, particularly its amphiphilicity, aggregation tendency, and molecular weight distribution, as these factors govern both solubility and interactions with purification matrices.

**Figure 3:**
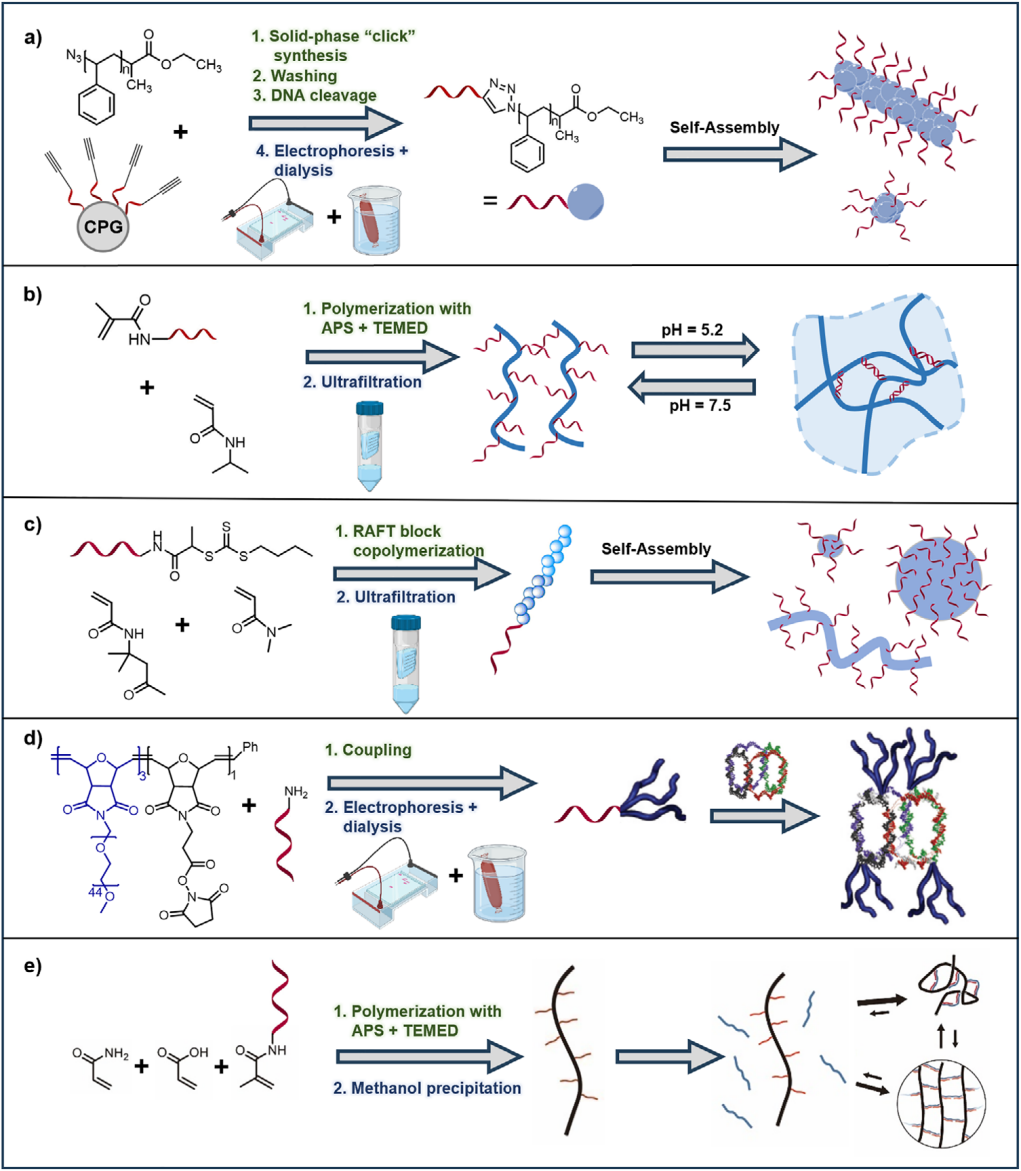
Synthesis and purification strategies of polymer‐DNA conjugates. a) PS‐DNA synthesis via solid phase coupling and purification using gel electrophoresis and extraction,^[^ ^131^ ^]^ b) PNIPAM‐DNA synthesis via *grafting from* and purification via ultrafiltration,^[^ ^53^ ^]^ c) P(DAAM‐DMA)‐DNA conjugate formation via RAFT polymerization using the *grafting from* method. After spin filtration the pure conjugate forms various morphologies,^[^ ^19^ ^]^ d) Formation of a three‐armed PEG‐DNA conjugate via NHS coupling. Purification was performed by gel electrophoresis and extraction. Parts reproduced with permission.^[^ ^137^ ^]^ Copyright 2012, American Chemical Society. e) Ultrahigh MW P(acrylamide‐*co*‐acrylic acid)‐DNA conjugate synthesis via *grafting from* and purification via methanol precipitation. Parts reproduced under the CC BY 4.0 license.^[^ ^138^ ^]^ Copyright 2023, The Authors. Synthesis and purification steps are highlighted in green and blue, respectively, for clarity. (Figure created with BioRender.com).

One example of these phase‐separating conjugates is polystyrene‐DNA (PS‐DNA).^[^ ^131^ ^]^ Phase separation between the DNA and polymer building blocks can pose challenges during synthesis and purification, as unreacted polymers may be trapped within the condensed phases, rendering ultrafiltration ineffective for purifying the products. To address this challenge, DNA was initially synthesized on solid support and equipped with an alkyne end group. Subsequently, PS polymer, containing an azide end group, was coupled to a solid‐supported DNA strand via copper(I)‐catalyzed azide‐alkyne cycloaddition (CuAAC) (**Figure** **3**a). The reaction mixture was then washed to remove unreacted polymer, cleaved, and purified. Given the differences in size‐to‐charge ratio of unreacted DNA and the conjugate, gel electrophoresis can serve as a useful purification method, capable of circumventing solubility issues and preserving higher‐order structures. This is achieved by applying a potential difference to drive the charged molecules or colloidal particles through a gel matrix, after which the conjugate band can be excised, extracted using a spin column, and desalted by dialysis (6–8 kDa MWCO).^[^ ^131^ ^]^ However, self‐assembly of uncharged polymer‐DNA conjugates into micellar structures may contain unreacted polymer chains. In such cases, migration behavior reflects aggregate properties rather than molecular composition. Similarly, fluorescent polymer‐DNA conjugates were synthesized using poly(9,9‐dioctylfluorene‐alt‐benzothiadiazole) (PFBT) as the polymer block, enabling the formation of photoactive, amphiphilic conjugates. Solid‐supported alkyne functionalized DNA (4.6 kDa) was coupled to azide PFBT (4.3 kDa) via CuAAC, followed by washing to remove unreacted polymer and other impurities. Since polymer removal occurs prior to cleavage, DNA impurities can be easily eliminated using ultrafiltration.^[^ ^74^ ^]^

Multiblock polymer‐DNA conjugates, such as poly(hexaethylene‐hexaethylene glycol)‐DNA (P(HE‐HEG)‐DNA), were synthesized via similar approaches, with the polymer block added step by step.^[^ ^133^ ^]^ Such multiblock conjugates are interesting as they can self‐assemble into a range of structures mediated by hydrophobic and other supramolecular interactions. Initially, the DNA block (5.8 kDa) was constructed using a DNA synthesizer, with phosphoramidite functionalized polymer monomers sequentially coupled to produce a 12‐unit long polymer block. The step‐by‐step coupling approach ensures precise definition of sequence and monodispersity. Furthermore, this strategy facilitates easier removal of small impurities due to the washing step after each coupling. At the end of synthesis, the solid supported conjugate (9.4 kDa) underwent washing to remove unreacted monomers, followed by cleavage and ultrafiltration (0.22 µm) to remove solid impurities. RP‐HPLC (C18) was performed using an elution gradient of 3–70% ACN to isolate the respective conjugate.^[^ ^133^ ^]^ The same synthesis procedure was used to create poly(9,9‐di‐*n*‐octylfluorenyl‐2,7‐diyl)‐DNA conjugates (PFO‐DNA) as dispersing agents to aid in solubilizing insoluble nanostructures, such as single‐walled nanotubes (SWNT).^[^ ^79^ ^]^ PFO‐phosphoramidite was synthesized, and solid‐phase synthesis performed to assemble a growing conjugate on the DNA block (DNA: 6.6 kDa, polymer: 5.3 kDa). Following cleavage and deprotection, AEC (HiTrap Q HP) was used to purify the crude conjugate. However, this method is only applicable when the conjugate is water‐soluble due to interactions with the charged resin of the column. In this case, a gradient of Tris‐HCl buffer and NaCl‐Tris buffer was used to elute the conjugate separately from the impurities. Subsequently, desalting of the conjugate was achieved via dialysis (2 kDa MWCO).^[^ ^79^ ^]^

Semiconducting polymer‐DNA conjugates offer a unique combination of the optoelectronic properties of the conjugated polymer with the programmable molecular recognition capability of DNA. To introduce a semiconductive conjugate capable of size‐controllable micelle formation, a poly[3‐(2,5,8,11‐tetraoxatridecanyl)thiophene]‐DNA conjugate (PTOTT‐DNA) was synthesized.^[^ ^139^ ^]^ The high amphiphilicity of the resulting conjugate makes it amenable to solid support synthesis using phosphoramidite monomers. PTOTT‐phosphoramidite (6.9 kDa) was coupled to the 5′ end of the solid‐supported DNA (7.7 kDa). After washing and cleavage, purification was performed by rapidly dissolving the solution in water and centrifuging, exploiting the good water solubility of the unreacted DNA to remain in the aqueous phase while the conjugate precipitated out of solution.^[^ ^139^ ^]^ Similar synthesis procedures can be performed to obtain polypropylene glycol‐DNA conjugates (PPG‐DNA). Denaturing PAGE (dPAGE) was employed for purification to prevent micellization or precipitation during isolation. Subsequently, the conjugates were filtered and desalted.^[^ ^140^ ^]^ To produce morphologies of biodegradable conjugates such as micelles, polycaprolactone‐DNA (PCL‐DNA) can be used. PCL is functionalized with multiple azide groups, leading to the formation of brush‐like PCL‐DNA conjugates through SPAAC. Due to the significant size difference between the conjugate and the unreacted DNA, ultrafiltration (50 kDa MWCO) was performed to achieve high purity.^[^ ^141^ ^]^

Poly(*N*‐isopropylacrylamide)‐DNA conjugates (PNIPAM‐DNA) are commonly used to introduce stimuli‐responsive materials exhibiting thermo‐sensitivity, enabling the creation of materials with switchable morphologies.^[^ ^52^ ^]^ These conjugates exhibit a lower critical solution temperature (LCST) in the physiological range of 30 °C to 35 °C, making them appealing for biomedical applications. PNIPAM solubilizes at temperatures below its LCST and agglomerates above. To manufacture and purify these conjugates, several strategies have been used. One approach involves using CuAAC to couple PNIPAM (5.3/10.4/19.8 kDa) to DNA, followed by RP‐HPLC (C18, gradient 5–70% ACN in TEAA) purification of the reaction mixture.^[^ ^142^ ^]^ In another study, PNIPAM (11.5 kDa) was coupled to solid‐supported DNA (5.2 kDa), washed, cleaved and purified by gel electrophoresis and extraction. PAGE was performed and the bands of interest were excised and redissolved to obtain the pure conjugate.^[^ ^52^ ^]^ To create brush‐like conjugates, DNA can be functionalized with DBCO groups via non‐natural nucleotides (DBCO‐2´‐deoxyuridine‐5´‐triphosphate, DBCO‐dUTP), allowing the grafting of PNIPAM using a SPAAC reaction. Given the high MW of the branched conjugate, excess polymer was removed via ultrafiltration (100 kDa MWCO).^[^ ^143^ ^]^ These brush‐like conjugates offer versatility in creating temperature‐responsive gels capable of swelling under physiological conditions. Another study demonstrated the creation of PNIPAM‐DNA conjugates with PNIPAM forming the backbone via free radical polymerization of NIPAM with acrydite‐functionalized DNA (Figure 3b). Excess monomers were easily removed via ultrafiltration (10 kDa MWCO).^[^ ^53^ ^]^ An innovative approach to use PNIPAM‐DNA is within a thrombin‐responsive pore, where the pore closes upon DNA hybridization due to a charge shift around the PNIPAM‐DNA conjugate, leading to polymer aggregation. The pore reopens upon removal of the complementary strand by thrombin. As in other studies, the conjugate is formed through *grafting to* and purified via ultrafiltration.^[^ ^144^ ^]^ PNIPAM‐RNA conjugates were obtained using an ATRP initiator attached to a Torula Yeast RNA backbone for photopolymerization. The obtained material was purified using dialysis in ice‐cold water to guarantee solubility. This study introduced temperature‐responsiveness to RNA‐based materials and can also provide degradation resistance to the RNA block.^[^ ^87^ ^]^

Poly(diacetone acrylamide‐dimethylacrylamide)‐DNA conjugates (P(DAAM‐DMA)‐DNA) can form a variety of self‐assembled architectures depending on polymer length and block ratios. Polymerization‐induced self‐assembly (PISA) was achieving using the *grafting from* strategy through RAFT polymerization with DNA containing a macro initiator (Figure 3c). This technique resulted in only small impurities or unreacted excess, which could easily be removed by ultrafiltration.^[^ ^19^ ^]^ Another strategy to obtain high yields of P(DAAM‐*b*‐DMA)‐DNA conjugates is the *grafting to* strategy. Here, the polymer is coupled through reactive groups to the DNA block in excess. AEC effectively purified the reaction solution. The reaction mixture was loaded onto a column, where charged molecules interacted with the charged resin, and uncharged molecules were washed away. After this, a gradient of NaCl solution was applied to elute the unreacted oligonucleotide as well as the conjugate from the column and separate them.^[^ ^88^ ^]^ RNA conjugates can also exhibit high amphiphilicity and undergo micellization when combined with hydrophobic polymers. This was demonstrated for the formation of poly(methyl acrylate)‐RNA (PMA‐RNA; PMA: 41 kDa; RNA: 6.3 kDa) by attaching an ATRP initiator to the RNA strand. After polymerization, unreacted small molecules could be removed by dialysis and precipitation in water.^[^ ^145^ ^]^

#### 3.1.3 Hydrophilic Polymers

The design of increasingly hydrophilic polymer‐nucleic acid conjugates has gained attention for stabilizing or programming self‐assembly onto nanoscale architectures such as DNA origami and smaller constructs like multiarm DNA prisms.^[^ ^69^, ^137^, ^146^ ^]^ Unlike amphiphilic conjugates, hydrophilic polymer conjugates do not spontaneously form distinct morphologies but can effectively stabilize other structures in solution. Additionally, hydrophilic polymer‐nucleic acid conjugates play a crucial role in creating self‐healing hydrogels, leveraging the complementarity and dynamic hybridization behavior of DNA strands to facilitate gel reconnection and reassembly after mechanical damage.^[^ ^17^ ^]^ Moreover, the polymer block of these conjugates can provide protection against nuclease degradation or enhance biocompatibility.^[^ ^137^ ^]^

The high water solubility and reduced non‐specific interactions of hydrophilic polymer‐nucleic acid conjugates, compared to their amphiphilic counterparts, strongly influence the choice of purification method. As these materials remain well‐dispersed in aqueous environments and interact only weakly with hydrophobic stationary phases, RP‐HPLC is often less suitable unless ion‐pairing agents are employed to improve retention. Instead, AEC and SEC are commonly used, as they allow purification under mild aqueous conditions. Ultrafiltration and dialysis are also frequently applied for buffer exchange and removal of excess reactants, offering gentle conditions that preserve structural integrity. The hydrophilic nature and reduced tendency for aggregation or non‐specific adsorption make these techniques particularly effective. However, due to their high solubility, precipitation‐based methods such as alcohol‐ or PEG‐induced precipitation are often inefficient or fail to achieve sufficient recovery.

To facilitate the self‐assembly of DNA prisms, poly(hexaethylene) phosphate conjugates with DNA were used (5.8 kDa; polymers with 1 to 12 units). Phosphoramidite‐modified monomers of hexaethylene phosphate were synthesized and coupled to solid‐supported DNA, allowing precise control over the formation and number of monomers constituting the polymer block. After coupling, washing, and cleavage, the mixture underwent purification using PAGE. The separated bands were excised and incubated in sterile water. Additionally, the sample underwent filtration, drying, and desalting by SEC (Sephadex G‐25).^[^ ^146^ ^]^ The same purification strategy was applied to synthesize three‐arm PEG‐DNA conjugates (Figure 3d). The objective was to prepare organized coatings on DNA architectures to stabilize the structure and enhance biocompatibility. A PEG_44_ macromonomer was polymerized (6.7 kDa) using ring‐opening metathesis polymerization (ROMP), coupled post‐polymerization to DNA (4.3 kDa), and purified following the aforementioned procedure.^[^ ^137^ ^]^ In another approach, polymers were first synthesized containing phosphoramidite‐modified nucleic acids to construct brush‐like conjugates, with the oligonucleotides forming the side chains. These conjugates were used to create programmable arbitrary polymer routings on DNA origami, with the potential to form optical wires based on the conjugated properties of the polymer. Poly(2,5‐dialkoxy)paraphenylene vinylene‐tert‐butyldiphenylsilyl conjugates with DNA (P(APPV‐TBPDS)‐DNA) were synthesized by initially loading the polymer onto 2‐deoxythymidine (dT) and conducting solid‐phase DNA synthesis. Following cleavage and deprotection, the conjugates underwent purification using SEC (PD‐10) and elution with TEAA buffer.^[^ ^147^ ^]^ In another approach, water‐soluble polymer‐DNA conjugates were purified using AEC for a variety of conjugates (polymers: PDMA, 10, 20, and 48 kDa; PNIPAM 20 kDa; poly(hydroxyethyl acrylate) (PHEA) 20 kDa; P(DAAM‐*b*‐DMA) 27 kDa or poly(oligo(ethylene glycol) methacrylate) (POEGMA) 20 kDa). These conjugates demonstrated annealing capability on DNA origami surfaces to create patterns with nanometer resolution. After loading the reaction mixture onto the chromatography column, unreacted polymer was removed via washing, and a NaCl gradient was introduced to partially dissociate charged molecules from the column. This method facilitated the recovery of unreacted DNA and yielded excellent quantities (60%) of the obtained conjugate.^[^ ^69^, ^88^ ^]^ PHEA was utilized to create RNA conjugates by equipping the RNA strand with an ATRP initiator and conducting photoinduced polymerization. Due to minimal reaction impurities, dialysis was employed for purification.^[^ ^145^ ^]^ For the creation of polymer‐DNA hydrogels, star shaped conjugates can be used. In this method, DNA strands (4.9 kDa) undergo coupling via NHS chemistry to a 4‐arm PEG polymer star (40 kDa). With respect to the size difference between unreacted DNA and the conjugate, SEC (Superdex 200) was used, yielding the pure conjugate.^[^ ^17^ ^]^

PEG‐RNA conjugates were created by the functionalization of PEG polymers (0.4/1/4 kDa) with phosphoramidite and coupling to solid‐supported RNA (1.7 kDa). After coupling, washing, and cleavage, RP‐HPLC (C4, gradient: 0.8 to 32% ACN in TEAA) was employed.^[^ ^148^ ^]^ The same purification technique was used post‐synthesis of thiol‐modified PEG and RNA via a sulfhydryl exchange reaction.^[^ ^149^ ^]^ For constructing brush‐like conjugate materials using the *grafting from* technique, a RNA/DNA backbone with an ATRP initiator and photoactive group was used. After synthesizing the RNA/DNA backbone via solid‐phase synthesis, polymerization of oxopentanoate ethyl methacrylate (OEMA_500_) was performed using photopolymerization to form the brush‐like conjugate. Utilizing the *grafting from* technique, only small molecule impurities remained, which could be readily removed by ultrafiltration (100 kDa MWCO).^[^ ^132^, ^150^ ^]^ In another study, POEMA_500_‐RNA (torula yeast) brush conjugates were formed similarly by attaching an ATRP initiator to the RNA backbone and performing photo‐catalyzed polymerization. However, the product was purified using RP‐HPLC (C18).^[^ ^87^ ^]^ Expanding the polymer library, linear PEG‐methacrylate‐pOEOMA_475_‐RNA, pOEOMA_300_‐*co*‐MEO_2_MA‐RNA, and pOEOMA_475_‐*co*‐DMAEMA‐RNA conjugates were prepared, where the polymer block acted as a protector against nucleases and facilitated cellular internalization. These polymers, with an average MW of 20 kDa, were synthesized and coupled via CuAAC to short interfering RNA (siRNA) (11.8 kDa). After coupling, ultrafiltration (30 kDa MWCO) was used to remove catalyst and unreacted components.^[^ ^36^ ^]^

#### 3.1.4 Charged Polymers

Conjugates of nucleic acids with charged polymers, both cationic and anionic, have been explored for a range of applications, including DNA sensors,^[^ ^151^ ^]^ immune stimulation,^[^ ^152^ ^]^ intracellular delivery,^[^ ^153^ ^]^ and supramolecular hydrogels.^[^ ^138^ ^]^ Charged polymers extend the physicochemical properties of nucleic acid conjugates, introducing pH‐responsive polymers with acidic or basic groups.^[^ ^154^ ^]^ For instance, π‐conjugated polymers, widely applied in sensor technologies, face challenges in water solubility, which can be overcome by incorporating charged side chains.^[^ ^151^ ^]^ The carboxyl groups of anionic polymer scaffolds, similarly to the amino groups of cationic polymers, facilitate the introduction of orthogonal groups for the spatial display of bioactive molecules.^[^ ^152^ ^]^ High MW poly(acrylamide‐co‐acrylic acid) demonstrates biocompatibility and responsiveness to methanol, making it suitable for coculture with cells.^[^ ^138^, ^155^ ^]^ Cationic polymers, with the capacity to cross cell membranes, have been instrumental in delivering therapeutic molecules into cells.^[^ ^156^ ^]^ Few studies have explored charged polymer conjugates with nucleic acids, all of which were conducted in aqueous solution. This limited study may stem from electrostatic repulsion between anionic nucleic acids and anionic polymers, resulting in low reaction yields.^[^ ^41^ ^]^ Conversely, cationic polymers are electrostatically attracted,^[^ ^157^, ^158^, ^159^, ^160^ ^]^ which can hinder targeted conjugation of reactive sites in each block, also leading to low yields and challenging purification processes. Moreover, the ratio of cationic polymer to anionic nucleic acids, along with the high density of cationic units within the polymer, may contribute to potential aggregation or precipitation during synthesis and purification.^[^ ^41^, ^161^ ^]^ Currently, most studies focus on cationic polymers, while the nucleic acids are conjugated non‐covalently based on electrostatic interactions. Among the available purification methods, ultrafiltration^[^ ^151^, ^152^ ^]^ and PEG purification by selective precipitation^[^ ^153^ ^]^ have been the most commonly employed techniques.

As an illustration of anionic polymer‐DNA conjugates, poly(*p*‐phenyleneethynylene) (PPE) was used, with the π‐conjugated polymer exhibiting amplified fluorescence properties for DNA detection. PPE (MW 13 kDa) was conjugated with two carboxylic acids as end groups to DNA (MW 4.8 kDa) using carbodiimide chemistry at a 5‘‐end amine group.^[^ ^151^ ^]^ Sulfonate ions were introduced as side chains to enhance the water solubility of PPE, enabling solution chemistry, and DNA was added in excess to ensure complete reaction of all PPE. The resulting PPE‐DNA conjugate (MW 22 kDa) was purified through ultrafiltration (MWCO 10 kDa) to selectively remove free DNA. In the study, anionic polymer‐DNA conjugates were obtained via the *grafting to* method. Conversely, poly(acrylamide‐*co*‐acrylic acid)‐DNA conjugates (MW 3 MDa) were synthesized as 3D cell‐culture matrices by copolymerizing acrylamide, sodium acrylate and acrylamide‐functionalized DNA in the presence of ammonium persulfate (APS) and tetramethylethylenediamine (TEMED) as polymerization initiators (Figure 3e).^[^ ^138^ ^]^ Methanol precipitation was conducted to remove cytotoxic monomers, which could be harmful when incubated with cells.

To prepare a cationic polymer‐DNA conjugate, a photocaging strategy was used to facilitate in situ ATRP of the photocaged monomer 4‐(methacryloyloxytris‐(ethoxy)ethylcarba‐moyloxypropanyl)‐3‐nitrobenzoic acid (MCNB) on a DNA origami macroinitiator.^[^ ^153^ ^]^ MCNB, an anionic monomer with a carboxyl group, can be transformed into 2‐aminotris(ethoxy)ethylmethacrylate (AEMA), a cationic monomer with an amine group, under 365 nm UV irradiation. This transformation occurs as the UV‐sensitive carbamate cages are cleaved, exposing the basic amine groups. Similarly, anionic P(MCNB), formed on the DNA origami macroinitiator by a *grafting from* strategy, can be transformed into the cationic polymer P(AEMA). PEG purification was used to remove unreacted monomers as well as other impurities. For the direct conjugation of nucleic acids and cationic polymers, aiming to alleviate issues such as precipitation and aggregation during synthesis and purification, the ratio and density of cationic units present in polymers are crucial for obtaining cationic polymer‐nucleic acid conjugates of high purity.^[^ ^41^, ^161^ ^]^ Specifically, the ratio of cationic polymers to oligonucleotides should be kept as low as possible to ensure successful conjugation and prevent potential precipitation during synthesis. Additionally, the density of cationic units within polymers should be considered, as excessively high density may result in an inseparable mixture of conjugates, oligonucleotides, and cationic polymers that cannot be effectively purified further.

### 3.2 Peptide‐Nucleic Acid Conjugates

#### 3.2.1 General

Peptide‐nucleic acid conjugates have been investigated for various applications such as gene sequencing,^[^ ^162^ ^]^ self‐assembly,^[^ ^54^, ^163^ ^]^ protein mimics,^[^ ^164^ ^]^ hydrogels,^[^ ^165^ ^]^ enhanced cellular uptake by introducing CPPs or cell recognition motifs such as RGD,^[^ ^22^, ^166^ ^]^ and immune stimulation.^[^ ^23^ ^]^ While nucleic acids provide unparalleled programmability and sequence specificity,^[^ ^164^ ^]^ they lack diverse chemical functionalities.^[^ ^167^ ^]^ Conjugation to peptides therefore enables the combination of the programmable molecular recognition of nucleic acids with the structural and functional diversity of peptides, making peptide‐nucleic acid conjugates a highly versatile class of hybrid biomaterials. Peptides offer versatile biological functions, tunable charge and hydrophobicity, and synthetic flexibility.^[^ ^30^, ^167^ ^]^ However, the disparate physicochemical properties, including MW, charge, polarity, and acidity, induced by the sequence and length of the two building blocks pose challenges in the conjugation and purification of these conjugates,^[^ ^168^ ^]^ particularly regarding their polarity and charge characteristics.

Purification of peptide‐nucleic acid conjugates requires special consideration of their amphiphilicity and charge balance. Highly charged or amphiphilic conjugates may form aggregates or precipitate during purification, particularly when one of the components (e.g., cationic peptides) interacts electrostatically with the negatively charged phosphate backbone. Such behavior complicates both chromatographic and solvent‐based separation. The most widely employed purification method is RP‐HPLC,^[^ ^30^, ^164^, ^168^, ^169^, ^170^, ^171^, ^172^, ^173^, ^174^, ^175^ ^]^ which provides high resolution and reproducibility through hydrophobic interaction with the stationary phase. However, method development is often required to optimize gradients and mobile‐phase additives such as ion‐pairing reagents (e.g., TEAA or TEA/HFIP), as the balance of hydrophilic and hydrophobic domains influences retention and recovery. Moreover, strongly amphiphilic or aggregated conjugates may yield broad peaks or incomplete elution.

Alternative purification methods include PAGE,^[^ ^175^, ^176^ ^]^ dialysis,^[^ ^42^, ^54^ ^]^ ultrafiltration,^[^ ^30^, ^172^, ^174^ ^]^ gravity SEC,^[^ ^172^, ^177^, ^178^ ^]^ and AEC.^[^ ^37^, ^58^ ^]^ Each offers specific benefits: PAGE enables high‐resolution separation of small or charge‐differentiated conjugates; SEC and ultrafiltration provide gentle, low‐pressure methods for desalting and buffer exchange; and AEC separates species based on charge density, particularly useful for oligonucleotide‐rich conjugates. Nevertheless, these methods generally suffer from limited selectivity compared to RP‐HPLC.

In solid‐phase synthesis approaches, purification is often integrated into the synthesis workflow, as unreacted components and side products can be efficiently removed through repeated washing steps using aqueous or organic solvents.^[^ ^42^, ^54^, ^163^, ^169^ ^]^ This strategy significantly reduces purification complexity and loss of product, especially for conjugates that are otherwise prone to aggregation or precipitation during liquid‐phase workup.

While robust RP‐HPLC remains the method of choice for most peptide‐nucleic acid conjugates, complementary low‐pressure techniques such as SEC, ultrafiltration, or PAGE are often used for enhancing purity or desalting. For conjugates with extreme hydrophobicity or strong electrostatic interactions, optimization of ionic strength, pH, and buffer composition is essential to maintain solubility during purification.

#### 3.2.2 Purification Strategies Following Solid‐Phase Synthesis

Strategies for synthesizing peptide‐nucleic acid conjugates typically involve solid‐phase stepwise synthesis and fragment conjugation.^[^ ^59^ ^]^ In solid‐phase stepwise synthesis, akin to the *grafting from* strategy for polymers, nucleic acids and peptides, or vice versa, are sequentially elongated on identical solid support, necessitating specific protecting groups for side chains and constraining the length of nucleic acids and peptides. In contrast, fragment conjugation, akin to the *grafting to* strategy for polymers, involves synthesizing nucleic acids and peptides individually, followed by the conjugation of the two fragments in either solid or liquid phase. This method uses conventional protecting groups and imposes no restrictions on the length of the two building blocks, but necessitates careful consideration of the solubility and charge of the building blocks.^[^ ^59^ ^]^

Using solid‐phase stepwise synthesis, peptide‐DNA conjugates were synthesized for therapeutic applications.^[^ ^179^ ^]^ A branched trifunctional linker was conjugated to PEG‐PS resin with an amino group in a reversible way. Subsequently, an insulin‐like growth factor 1 (IGF1) d‐peptide analogue was synthesized via Fmoc chemistry, starting from the protected amino group of the linker. This was followed by DNA synthesis using phosphoramidite chemistry, starting from the protected OH group. Using mild deprotection conditions, cleavage was conducted in aqueous ammonia. The resultant conjugate underwent purification via RP‐HPLC (C18, gradient of 4.5–58.5% ACN in TEAA). In another work, peptide‐DNA conjugates with up to four AAs (tetrapeptides),^[^ ^180^ ^]^ incorporating most natural AAs except arginine and histidine, were synthesized from 3′‐end CPG‐supported DNA hexamers and octamers with an amino group terminally protected at their 5′end. Tryptophan and lysine were sequentially elongated at the 5′‐end of DNA through Fmoc chemistry. After Fmoc deprotection, RP‐HPLC or AEC was used to remove impurities. Similarly, a series of CPP‐DNA conjugates containing two to eight amine and guanidinium moieties were prepared for intercellular delivery of oligonucleotides.^[^ ^22^ ^]^ Initially, PEG‐PS resin‐supported peptides with two, four, and eight lysine residues were synthesized employing Boc chemistry. After deprotection, DNA sequences were elongated in a DNA synthesizer, followed by cleavage from resins. The lysine‐DNA conjugates underwent filtration, concentration, and desalting via gravity SEC (NAP‐10), followed by purification through semipreparative HPLC. Homoarginine‐DNA conjugates were obtained by introducing guanidinium groups into lysine‐DNA conjugates, followed by desalting using gravity SEC (NAP‐5).

**Figure 4:**
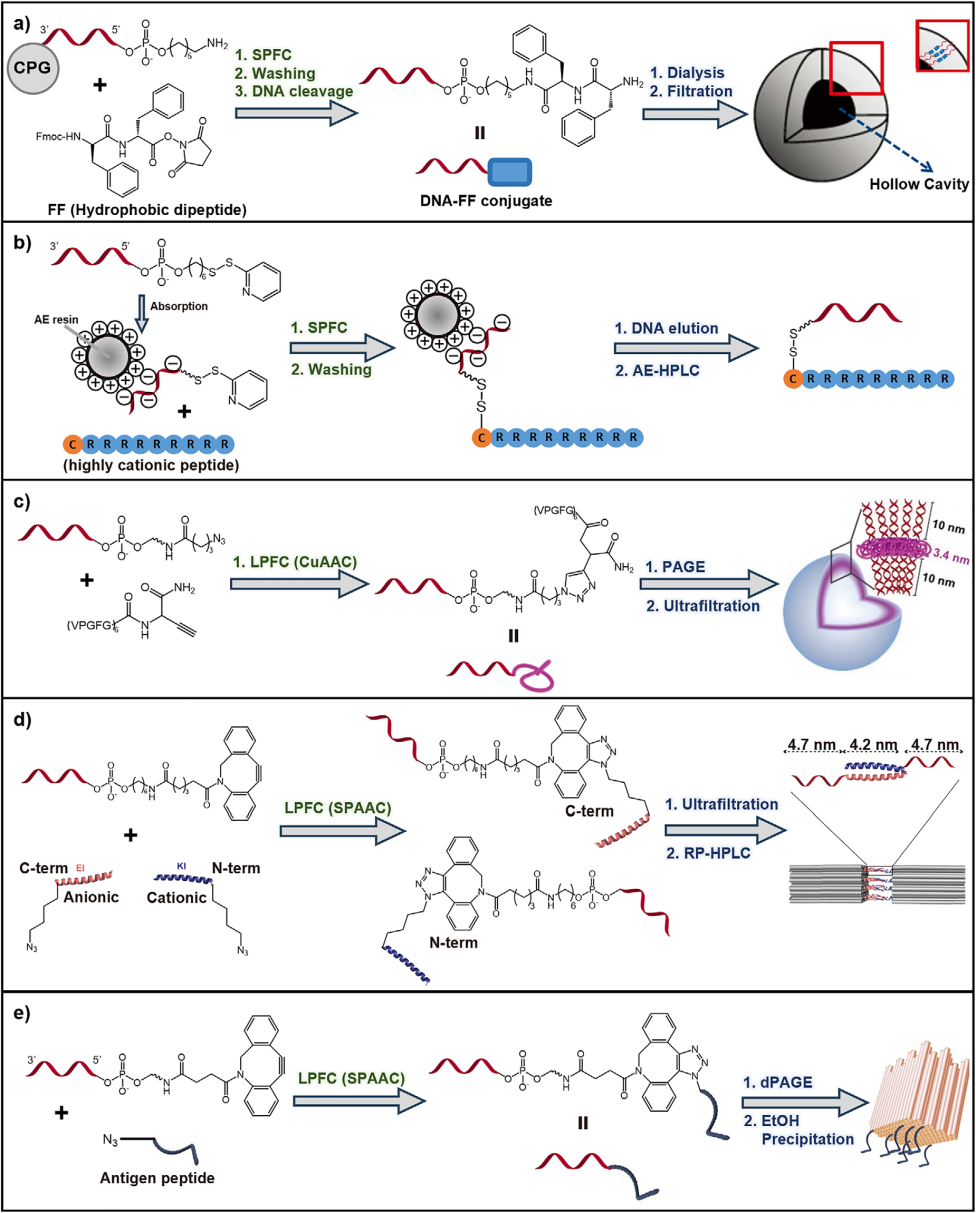
Synthesis and purification strategies of peptide‐DNA conjugates. a) FF‐DNA conjugate synthesis employing SPFC, followed by purification using dialysis and membrane filtration. Parts reproduced with permission.^[^ ^42^ ^]^ Copyright 2012, Royal Society of Chemistry. Permission conveyed through Copyright Clearance Center, Inc. b) Synthesis of arginine rich peptide‐DNA conjugates via SPFC, followed by purification using AEC.^[^ ^37^ ^]^ c) ELP‐DNA conjugate synthesis via LPFC through CuAAC, followed by purification through PAGE and ultrafiltration. Parts reproduced adapted with permission.^[^ ^176^ ^]^ Copyright 2021, Royal Society of Chemistry. Permission conveyed through Copyright Clearance Center, Inc. d) Charged peptide‐DNA conjugate synthesis via LPFC using SPAAC, followed by purification using ultrafiltration and RP‐HPLC. Parts reproduced with permission.^[^ ^30^ ^]^ Copyright 2019, American Chemical Society. e) Antigen peptide‐DNA conjugate synthesis via LPFC using SPAAC, followed by purification using dPAGE and ethanol precipitation. Parts reproduced adapted with permission.^[^ ^23^ ^]^ Copyright 2024, Springer Nature. Synthesis and purification steps are highlighted in green and blue, respectively, for clarity. (Figure created with BioRender.com).

Using the fragment conjugation strategy, peptide‐DNA conjugates were synthesized through solid‐phase fragment condensation (SPFC).^[^ ^169^ ^]^ This method adds larger fragments rather than adding one building block at a time and affords relatively high yields. Initially, a 3′‐end CPG‐supported oligonucleotide fragment with an amino group on its 5′‐end was coupled to 1,6‐diisocyanatohexane. Following this, washing with ACN was conducted to remove excess diisocyanate, after which partially protected peptide fragments with an amino group were introduced to yield 3′‐end CPG‐supported peptide‐oligonucleotide conjugates. Subsequent steps involved cleavage from the CPG support and deprotection with aqueous ammonia, followed by purification using RP‐HPLC with a gradient of 7–70% ACN in TEAA. TEAA buffer served as an ion pairing agent.^[^ ^168^ ^]^ Oligonucleotide fragments were synthesized using cyanoethylphosphoramidite chemistry, while peptide fragments were prepared using Fmoc chemistry. Similarly, the SPFC strategy was used to conjugate a 3′‐end CPG‐supported oligonucleotide fragment with an amino group on its 5′‐end to hydrophobic diphenylalanine (FF),^[^ ^42^, ^54^ ^]^ ditryptophan (WW),^[^ ^54^, ^163^ ^]^ and the amyloid beta peptide motif (16–21) (KLVFFA)^[^ ^54^ ^]^ for self‐assembly studies (**Figure** **4**a). Following washing to remove unreacted peptides and other side products, the CPG support and protecting groups were removed using ammonia. Subsequent steps involved dialysis (MWCO 2 kDa)^[^ ^42^, ^54^ ^]^ against MilliQ water or lyophilization and dissolution^[^ ^163^ ^]^ in nuclease‐free water, followed by filtration through a 0.45 µm membrane^[^ ^163^ ^]^ to remove non‐polar impurities. Cationic peptide‐nucleic acid conjugates, owing to the opposite charges of the two building blocks, may precipitate during reaction, posing challenges in purification. For instance, ASOs were conjugated to highly cationic arginine‐rich peptides (with a maximum of 9 arginine residues) through on‐resin fragment conjugation (a variant of SPFC) (Figure 4b).^[^ ^37^ ^]^ This method involved binding ASOs with a pyridine sulfenyl‐activated thiol group at the 5′‐end to an anion exchange (AE) resin column through electrostatic interaction. Then, cysteine residue‐containing arginine‐rich peptides were added, reacting with the absorbed oligonucleotides through the formation of disulfide bonds. Following washing with water to remove unreacted cationic peptides, the absorbed conjugates were eluted. An oligonucleotide purification cartridge was further used for desalting, followed by purification via AEC‐HPLC with a 0–80% gradient of 1 m NaCl in water.

#### 3.2.3 Purification Strategies Following Liquid‐Phase Synthesis

Peptide‐oligonucleotide conjugates can also be achieved through liquid‐phase fragment conjugation (LPFC).^[^ ^175^ ^]^ This approach offers a practical alternative to solid supported methods that lowers the technical barrier and is suitable for sterically complex conjugates. For instance, a heterobifunctional linker, succinimidyl‐4‐(*N*‐maleimidomethyl)cyclohexane‐1‐carboxylate (SMCC), was used to conjugate DNA with an amino group to cysteine‐containing peptides. Initially, the NHS moiety of the SMCC linker was conjugated to the amine‐modified DNA, followed by conjugation of the maleimide moiety of the SMCC linker to the thiol group of cysteine‐containing peptides. Then, RP‐HPLC or PAGE was performed to remove unreacted peptides and other impurities. RP‐HPLC purification involved a gradient of 5–100% ACN, while for PAGE purification, target bands were excised, dissolved in gel elution buffer, and then passed through a 0.45 µm spin filter. Ethanol precipitation^[^ ^175^ ^]^ was performed to remove any stains such as SYBR Gold from PAGE purification. Following either RP‐HPLC or PAGE purification, a gravity SEC (NAP‐10) was used to remove excess salts. Likewise, conjugates of DNA with weakly hydrophobic elastin‐like polypeptides (ELPs) were prepared through LPFC for self‐assembly studies (Figure 4c).^[^ ^176^ ^]^ In this process, DNA with an azido group on its 5′ or 3′ end was conjugated to ELPs with an alkyne group through CuAAC reaction. Subsequently, PAGE purification was performed, wherein excised bands were dialyzed against water and concentrated by ultrafiltration (MWCO 3 kDa).

Aptamers with a 5′‐end amino group were conjugated to peptide amphiphiles (PAs), which consist of a hydrophobic tail and a peptide sequence with charged residues to ensure solubility in the reaction buffer, through a heterobifunctional linker of dibenzocyclooctyne‐sulfo‐*N*‐hydroxysuccinimidyl ester (DBCO‐sulfo‐NHS).^[^ ^172^ ^]^ Initially, the NHS moiety of the DBCO‐sulfo‐NHS linker was conjugated to the amine‐modified aptamer, then the DBCO moiety of the DBCO‐sulfo‐NHS linker to PAs via SPAAC. Excess PAs were removed through ultrafiltration (MWCO 3 kDa), followed by SEC (NAP‐5) purification to desalt. RP‐HPLC purification was performed with a gradient of 4.5–90% ACN in TEAA. In another study, DNA of different lengths (19 and 46 bases) with an amino group on its 3′ or 5′‐end was conjugated to the azidolysine residue of FF dipeptides (Fmoc‐FF‐(PEG)_2_‐azidolysine‐NH_2_) through a DBCO‐sulfo‐NHS linker for morphological studies of self‐assembly.^[^ ^174^ ^]^ (PEG)_2_ units were inserted into FF dipeptides to increase solubility in the reaction buffer. After the reaction, ultrafiltration (MWCO 3 kDa) was conducted, followed by RP‐HPLC (C18) purification. These conjugates can self‐assemble into nanofibers and can be further cross‐linked in the presence of DNA, making them attractive candidates for the synthesis of engineered synthetic cytoskeletons for the bottom‐up construction of artificial cells.^[^ ^73^ ^]^

To achieve coiled‐coil self‐assembly of peptide‐DNA conjugates and further application in DNA origami, DNA with an amino group on its 5′‐end was coupled to the azidolysine residue on the C‐terminus of anionic domain‐containing peptides and the N‐terminus of cationic domain‐containing peptides separately through DBCO‐sulfo‐NHS (Figure 4d).^[^ ^30^ ^]^ Initially, the NHS moiety of the DBCO‐sulfo‐NHS linker was conjugated to the amine‐modified DNA, then the DBCO moiety of the DBCO‐sulfo‐NHS linker to azide‐modified peptides via SPAAC. DNA and cationic domain‐containing peptides were reacted at a 1:1 ratio to avoid electrostatic aggregation. After the reaction, ultrafiltration (MWCO 3 kDa) was conducted and the conjugate purified through RP‐HPLC (C18) with a gradient of 10–100% methanol in TEAA. In another study, a series of cationic peptide‐DNA conjugates were synthesized through LPFC by coupling DNA with an amino group on its 5′‐end to thiol‐modified peptides using the SMCC linker.^[^ ^178^ ^]^ Cationic peptides, composed of five residues, were variable to the cationic AAs lysine, ornithine, histidine and arginine, the hydrophobic AA tryptophan, and alanine as a spacer. Conjugates were purified through a NAP‐5 column, eluting with water to remove excess peptides. As aforementioned, it is extremely difficult to synthesize and purify highly cationic peptide‐nucleic acid conjugates in the liquid phase due to precipitation. Oligonucleotides with a pyridine sulfenyl‐activated thiol group at its 5′‐end were conjugated to highly cationic peptides with a thiol‐containing cysteine residue derived from the HIV‐Tat protein via LPFC through the formation of a disulfide bond.^[^ ^58^ ^]^ To avoid precipitation, high salt concentration of KCl and ACN of up to 40% were used to help solubilization during the coupling reaction. AE‐HPLC was performed to remove excess cationic peptides, using a gradient from 20–60% of 0.65 M KCl in 40% (v/v) ACN. Conjugates were precipitated into ethanol to desalt. For the synthesis of conjugates of highly cationic peptides with nucleic acids, denaturing conditions such as salts at high concentration and formamide addition are important.^[^ ^59^ ^]^ Conjugates with therapeutic peptides like antigens play an important role in their application in biomedical science. For instance, DNA with an amino group at its 5′‐end was conjugated to an azide‐modified neoantigen peptide through LPFC via the DBCO‐sulfo‐NHS linker and incorporated into DNA origami for immune stimulation (Figure 4e). 8% dPAGE and ethanol precipitation were performed for purification.^[^ ^23^ ^]^

### 3.3 Protein‐Nucleic Acid Conjugates

#### 3.3.1 General

Protein‐nucleic acid conjugates have gained significant attention due to their diverse applications in immune stimulation,^[^ ^23^ ^]^ intracellular sensing,^[^ ^181^, ^182^ ^]^ Janus nanoparticle formation,^[^ ^183^ ^]^ and hierarchical assembly.^[^ ^184^ ^]^ Proteins, ranging from enzymes and antibodies to engineered scaffolds, offer vast functional diversity within biological systems,^[^ ^185^, ^186^, ^187^ ^]^ enabling the combination of catalytic, binding, and signaling functions with the programmability of nucleic acids. Enzymes catalyze essential biochemical reactions,^[^ ^188^, ^189^, ^190^ ^]^ antibodies provide highly specific molecular recognition,^[^ ^191^, ^192^ ^]^ and the integration of nucleic acids enables sequence‐controlled self‐assembly. Naturally occurring protein‐nucleic acid conjugates,^[^ ^193^ ^]^ such as DNA‐topoisomerase 1 (TOP1) cross‐links, illustrate the functional potential of covalent protein‐nucleic acid connections, which are now increasingly exploited through chemical synthesis approaches analogous to the *grafting to* strategy for polymers.^[^ ^194^ ^]^

However, the chemical synthesis of protein‐nucleic acid conjugates typically produces heterogeneous mixtures containing unreacted components and conjugates with variable numbers or locations of attached nucleic acids. Such heterogeneity can compromise hybridization efficiency, biological function, or reproducibility, underscoring the need for efficient and selective purification methods.^[^ ^51^, ^195^ ^]^ Unconjugated proteins may also influence potential delivery efficiency and self‐assembly behavior.^[^ ^196^ ^]^ The purification process is particularly challenging because proteins differ vastly in MW (5–200 kDa or higher), isoelectric point, and tertiary structure stability, whereas nucleic acids are polyanionic, relatively rigid, and hydrophilic. Conjugation can therefore alter charge distribution, polarity, and hydrodynamic radius in complex ways, leading to difficulties in separating conjugates from unreacted species and in maintaining protein integrity during purification.

The yield and purity of protein‐nucleic acid conjugates depend on several factors: protein MW, the stoichiometric ratio of nucleic acid to protein, the number of solvent‐exposed reactive residues (e.g., lysines, cysteines), and the overall conjugation efficiency. Proteins exhibit a wide range of MWs, spanning from 5 kDa to over 200 kDa, while DNA MWs range from 6–30 kDa for single‐stranded DNA (20–100mer) to larger entities like plasmids and DNA origami structures.^[^ ^189^, ^190^ ^]^ Large proteins can accommodate multiple conjugation sites, often yielding polydisperse products, whereas site‐specific conjugation strategies aim to control stoichiometry and preserve functionality. In spherical nucleic acids (SNAs), for example, tens to hundreds of DNA strands can be attached per protein,^[^ ^181^, ^182^, ^197^, ^198^ ^]^ typically resulting in heterogeneous but functionally adequate assemblies purified by size‐based methods such as ultrafiltration or SEC.^[^ ^24^, ^182^, ^197^ ^]^ SNAs find usage in intracellular delivery and self‐assembly studies. In contrast, antibody‐DNA conjugates or therapeutic constructs require precise stoichiometry and structural uniformity, commonly achieved through site‐directed coupling and charge‐based purification such as AEC.^[^ ^24^, ^199^ ^]^ Given their potential clinical applications, obtaining homogeneous antibody‐DNA conjugates with predictable therapeutic indices is crucial.^[^ ^24^ ^]^

Methods for synthesizing protein‐nucleic acid conjugates often involve site‐selective (residue‐specific) coupling, targeting specific AA residues over others, or site‐specific coupling, which aims at single occurrences of particular AAs (e.g., non‐catalytic cysteine residues).^[^ ^194^ ^]^ Commonly selected AAs for coupling include lysine and cysteine, chosen based on considerations of abundance and reactivity, as well as methionine, tyrosine, serine, and genetically encoded AAs.^[^ ^200^ ^]^ Classical synthesis methods, such as site‐selective coupling, often result in random DNA labelling of proteins, resulting in heterogeneous products in terms of stoichiometry, which can impede their function and yield.^[^ ^47^, ^201^, ^202^ ^]^ Despite this limitation, the single‐step process and ease of purification make classical methods attractive for researchers. Modern methods aim to produce more homogeneous products, but they too face limitations, such as the requirement for distinct microenvironments for selective modification, such as the existence of metal‐binding sites^[^ ^203^ ^]^ and proximal reactive residues,^[^ ^194^ ^]^ or the need for multiple‐step synthesis in genetic manipulation of proteins, which can lead to low expression levels.^[^ ^194^ ^]^

Purification of protein‐DNA conjugates generally exploits differences in size (as influenced by the MW change induced by conjugated DNA), charge (resulting from the number of conjugated DNA strands and the distribution of surface‐reactive AA residues), and polarity (affected by the number of present conjugated hydrophilic DNAs). Ultrafiltration and SEC are the most frequently used techniques for size‐based separation and desalting.^[^ ^24^, ^181^, ^182^, ^196^, ^197^, ^198^, ^204^, ^205^ ^]^ They offer gentle, aqueous conditions that preserve protein conformation but provide limited resolution for conjugates with similar MW. AEC leverages the strong anionic character of nucleic acids to separate conjugates of different charge densities, proving particularly useful for conjugates with varying DNA load or for antibody‐DNA conjugates where precise separation is required.^[^ ^24^, ^199^, ^201^ ^]^ Affinity‐based methods, including biotin‐streptavidin interactions, His‐tag or Ni‐nitrilotriacetic acid (NTA) affinity purification, and magnetic bead‐based displacement, provide targeted selectivity but require engineered binding sites or affinity tags.^[^ ^196^, ^206^ ^]^ In contrast, polarity‐based purification of protein‐nucleic acid conjugates is less common and is typically reserved for cases where a significant polarity difference exists between the nucleic acid and protein components, such as for insulin. This method is employed when more conventional techniques have proven ineffective.^[^ ^199^ ^]^

Each purification approach has inherent limitations. Ultrafiltration and SEC cannot fully resolve heterogeneous conjugates differing only in the number of attached nucleic acids, while AEC may induce partial denaturation if ionic strength or pH is not carefully optimized. Furthermore, proteins’ sensitivity to organic solvents, extremes of pH, or ionic detergents constrains the range of usable chromatographic conditions. Thus, purification strategies are typically multistep, combining orthogonal methods, for instance, ultrafiltration or SEC for desalting, followed by AEC or affinity purification for selective enrichment of desired conjugates.

#### 3.3.2 Enzymes

Enzymes, as globular proteins, possess active sites that catalyze physiologically important functions with exceptional efficiency, with their size varying from less than 100 to over 2000 AA residues.^[^ ^188^ ^]^ Enzyme‐nucleic acid conjugates find applications in designing enzyme cascades on DNA origami structures, leveraging the programmability of nucleic acids.^[^ ^24^ ^]^ Additionally, they are used in constructing SNAs for intracellular catalysis and self‐assembly studies.^[^ ^181^ ^]^

##### Purification Strategies Based on Size Differences

**Figure 5:**
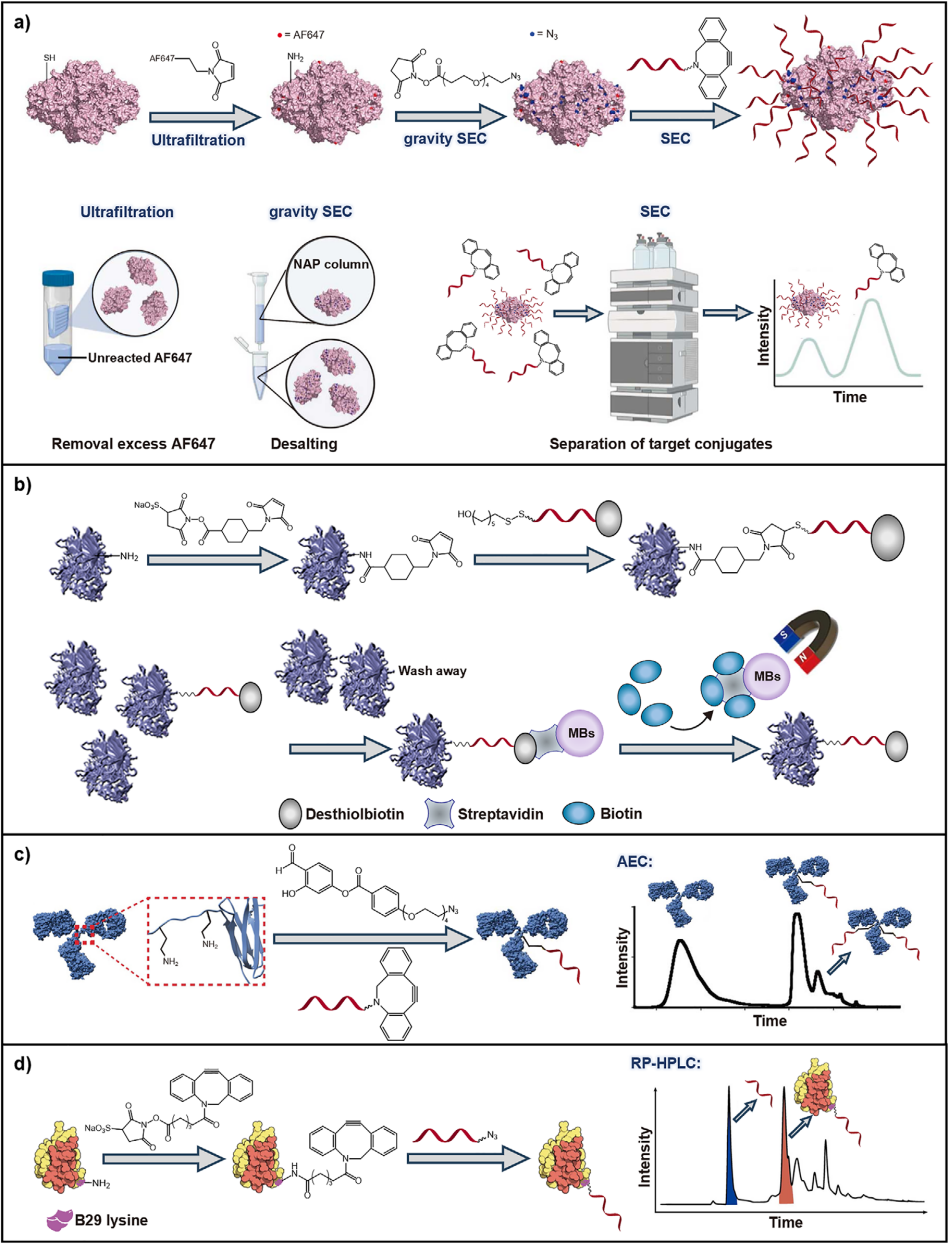
Synthesis and purification strategies of protein‐DNA conjugates. a) Schematic representation of β‐Gal‐DNA conjugate synthesis via SPAAC and subsequent purification using ultrafiltration, gravity SEC, and SEC separately. Reproduced adapted with permission.^[^ ^182^ ^]^ Copyright 2022, Springer Nature. b) Synthesis of invertase‐DNA conjugates through disulfide bond formation and purification using magnetic bead based biotin‐displacement.^[^ ^196^ ^]^ Reproduced adapted with permission.^[^ ^196^ ^]^ Copyright 2014, American Chemical Society. c) Belimumab‐DNA conjugate synthesis via SPAAC and purification using AEC. Reproduced adapted with permission.^[^ ^199^ ^]^ Copyright 2021, John Wiley and Sons. d) Synthesis of insulin‐DNA conjugates via SPAAC and purification using RP‐HPLC. Reproduced adapted under the CC BY 4.0 license.^[^ ^207^ ^]^ Copyright 2023, The Authors. Synthesis and purification steps are highlighted in green and blue, respectively, for clarity. (Figure created with BioRender.com).

To purify synthesis mixtures containing enzyme‐oligonucleotide conjugates and remove unconjugated oligonucleotides, ultrafiltration^[^ ^24^, ^181^, ^182^, ^196^, ^197^, ^198^, ^204^, ^205^ ^]^ is the most commonly used method. The choice of the MWCO for the filter is determined based on the MWs of the proteins and oligonucleotides involved.^[^ ^182^ ^]^ For example, in the case of β‐galactosidase (β‐Gal) (MW 446 kDa), which features both solvent‐accessible lysine and cysteine residues, and glucose oxidase (GOx) (MW 160 kDa), which has solvent‐accessible lysine residues,^[^ ^182^ ^]^ different DNA strands (MW 10–14 kDa) with a DBCO group at their 5′‐end were conjugated to β‐Gal and GOx using a NHS‐PEG_4_‐azide heterobifunctional linker through SPAAC and carbodiimide cross‐linking chemistry for SNA studies (**Figure** **5**a). The number of DNA strands conjugated per β‐Gal is ≈31 (with a theoretical maximum of 40), while specific information regarding GOx remains unavailable (with a theoretical maximum of 28). Unreacted DNA was removed through ultrafiltration (100 kDa MWCO) or SEC (ENrich SEC column) from β‐Gal‐SNAs. A MWCO of 100 kDa was used to ensure retention of the conjugate and removal of unconjugated DNA. When coupling proteins with smaller MWs such as horseradish peroxidase (HRP) (44 kDa), ultrafiltration with a smaller MWCO (e.g., 30 kDa) can be used. Similar procedures were employed for synthesizing i‐motif β‐Gal‐SNAs (with ≈30 conjugated i‐motif per β‐Gal) (MW of i‐motif 12 kDa) and T‐rich GOx‐SNAs (with ≈28 conjugated T‐rich DNA per GOx) (MW of T‐rich DNA 4 kDa).^[^ ^197^ ^]^ In these cases, ultrafiltration was performed using filters with MWCOs of 100 kDa for GOx‐SNAs and β‐Gal‐SNAs, respectively. Additionally, a series of β‐Gal‐SNAs (comprising T‐rich DNA with MW 10–14 kDa and G‐quadruplex‐forming DNA sequences with MW 6–12 kDa) with ≈30–37 conjugated DNA per β‐Gal were synthesized and purified by ultrafiltration (MWCO 100 kDa).^[^ ^181^ ^]^

In an alternative approach, DNA with a thiol group at its 3′‐end was conjugated to amine‐containing enzymes, including alcohol dehydrogenase (ADH) (MW 141–151 kDa), HRP (MW 44 kDa), and GOx (MW 160 kDa), using a succinimidyl 3‐(2‐pyridyldithio) propionate (SPDP) heterobifunctional linker, followed by purification via ultrafiltration (MWCO 30 kDa) to remove excess DNA (MW 17 kDa).^[^ ^205^ ^]^ Similarly, DNA with a thiol group at its 3′‐end was linked to amine‐containing GOx and HRP using a *N*‐[ɛ‐maleimidocaproyloxy] sulfosuccinimide ester (sulfo‐EMCS) linker, followed by purification through ultrafiltration (MWCO 30 kDa).^[^ ^204^ ^]^

##### Purification Strategies Based on Charge and Affinity Differences

While the ultrafiltration method effectively removes excess DNA from conjugates, it does not eliminate unconjugated enzymes, underscoring the importance of ensuring DNA excess and complete enzyme conjugation before purification. To achieve removal of unconjugated enzymes, an on‐bead biotin displacement method can used to purify enzyme‐DNA conjugates. 3′‐end desthiobiotin‐modified DNA (MW 11 kDa) with a thiol group at its 5′‐end was conjugated to invertase (MW 270 kDa) with amine groups using a sulfo‐SMCC linker, followed by ultrafiltration (MWCO 100 kDa) (Figure 5b).^[^ ^196^ ^]^ Desthiobiotin‐labelled conjugates were attached to streptavidin‐coated magnetic beads due to the high affinity of desthiobiotin to streptavidin, after which unconjugated invertase was washed away. Upon the addition of biotin, with a much higher affinity to streptavidin, desthiobiotin‐labelled DNA‐invertase conjugates were released from beads, yielding purified conjugates. This method can effectively remove excess DNA and unconjugated enzymes and can be applied to almost all proteins with solvent‐accessible amino groups, using the same procedure. However, while ultrafiltration, SEC, and magnetic bead‐based methods can remove unconjugated DNA, obtaining enzyme‐DNA conjugates with a defined number of conjugated DNA per enzyme may remain challenging.

When it is important to obtain conjugates with a defined number of DNA strands per enzyme, AEC can be used since nucleic acids are highly charged, and the method is suitable to separate differently charged species. This approach has been applied when assembling enzyme cascades on DNA origami. DNA bearing a thiol group at its 5′‐end was conjugated to amine groups of lysine residues on malic dehydrogenase (MDH) (MW 70 kDa) and glucose‐6‐phosphate dehydrogenase (G6pDH) (MW 100 kDa) using a SPDP linker.^[^ ^24^ ^]^ Ultrafiltration (MWCO 30 kDa) was used to remove excess DNA, and washing was conducted with high salt concentration buffer or detergent‐containing buffer to remove nonspecifically adsorbed DNA on the enzyme surface, resulting from electrostatic interactions. Despite the presence of multiple lysine residues on the enzyme surface, the enzyme‐DNA conjugates remained a mixture with varying numbers of conjugated DNA per enzyme. Subsequent AEC was conducted to separate unconjugated DNA and proteins from the conjugates, as well as to isolate conjugates with 1 to 4 conjugated DNA molecules per enzyme.

In the preceding methodologies, enzymes were initially conjugated to oligonucleotides, which could then be hybridized with large DNA scaffolds like DNA origami. However, further purification is necessary to remove any unhybridized conjugates. Another strategy is to link enzymes directly to DNA origami.^[^ ^208^ ^]^ For instance, streptavidin‐modified alkaline phosphatase (AP) (MW 94 kDa) and HRP were conjugated to biotin‐modified DNA origami bearing two hexahistidine affinity tags (His tags), exploiting the biotin‐streptavidin interaction.^[^ ^190^ ^]^ A cobalt‐based affinity purification was performed to separate unconjugated enzymes (HRP and AP) from enzyme‐conjugated DNA origami via the specific interaction between the His tags and a cobalt‐based immobilized metal affinity resin. Unconjugated enzymes were removed by washing, while enzyme‐DNA origami conjugates were later eluted from the resin. Another approach involved the preparation of S‐selective NADP+/NADPH‐dependent oxidoreductase (GRE2) (MW 38 kDa) variants, including with SNAP‐tag (GRE2‐Snap), Halo‐tag (GRE2‐Halo), and streptavidin‐binding peptide (SBP) tag (GRE2‐SBP), along with the reductase domain BMR of monooxygenase P450 BM3 (MW 64 kDa) with a Halo tag (BMR‐Halo), through a genetic fusion strategy.^[^ ^189^ ^]^ Subsequently, Halo‐ and Snap‐tagged enzymes were site‐selectively conjugated to DNA origami functionalized with suicide ligands (benzylguanine and chlorohexane groups) via interactions with their respective tags. GRE2‐SBP was linked to biotin‐functionalized DNA origami through biotin‐streptavidin interaction. Free‐flow electrophoresis (FFE) purification was then performed to separate unconjugated enzymes from enzyme‐conjugated DNA origami.

#### 3.3.3 Antibodies

Antibodies (immunoglobulins, Ig), typically weighing ≈150 kDa and measuring ≈10 nm in size, are composed of two heavy chains and two light chains, containing over 80 lysine residues which provide abundant sites for coupling via NHS chemistry.^[^ ^199^ ^]^ Antibody‐nucleic acid conjugates find extensive application in therapeutics, including targeted delivery and enhancing the circulatory half‐life of nucleic acids in the bloodstream, as well as in signal amplification for detection purposes.^[^ ^191^ ^]^ The synthesis of antibody‐nucleic acid conjugates via site‐directed mutagenesis and labelling yields more homogeneous conjugates with enhanced therapeutic efficacy.^[^ ^199^ ^]^ These conjugates are commonly purified through methods such as AEC,^[^ ^199^, ^201^, ^209^ ^]^ PAGE^[^ ^203^ ^]^ and immobilized metal‐ion affinity chromatography (IMAC).^[^ ^206^ ^]^

In one method, a template‐facilitated reaction was used to conjugate DNA, featuring an NHS ester group at its 3′‐end, to lysine residues of IgG antibodies (anti‐c‐Myc, anti‐FLAG M2, anti‐EGFR, and anti‐ß‐tubulin).^[^ ^203^ ^]^ Typically, guiding DNA, equipped with a tris‐NTA ligand at its 5′‐end, non‐covalently binds (coordinates) to IgG antibodies possessing a metal‐binding region (histidine‐rich cluster) on their constant Fc domain, in the presence of Cu(NO_3_)_2_, forming a Cu(II) complex. Subsequently, reactive DNA with an NHS ester group on its 3′‐end was added and hybridized with guiding DNA already bound to IgG antibodies, facilitating the reaction between NHS‐modified DNA and site‐selective lysine residues on IgG antibody surfaces due to their proximity. Following this, ultrafiltration (MWCO 3 kDa) was performed to concentrate samples, followed by PAGE purification, where targeted bands were excised and extracted by passive diffusion in EPPS buffer (4‐(2‐hydroxyethyl)‐1‐piperazinepropanesulfonic acid). While this templated method is suitable for the site‐selective conjugation of DNA to His6‐tagged proteins and wild‐type metal‐binding proteins like transferrin, proteins lacking metal‐binding sites are challenging to conjugate using this approach (except through specific tag incorporation via genetic fusion). Building upon the NTA‐modified DNA templated method, a peptide‐directed template method was developed to conjugate DNA, featuring a benzaldehyde group, to the amino group of lysine residues of IgG antibodies (Rituximab, Trastuzumab, Cetuximab,^[^ ^209^ ^]^ Panitumumab, and anti‐ß‐tubulin).^[^ ^201^ ^]^ Initially, a DNA strand functionalized with FC‐III peptide, serving as guiding DNA, non‐covalently attached to the Fc domain of IgG antibodies. The FC‐III peptide, a cyclic peptide, exhibits specific binding to certain domains of proteins. Subsequently, reactive DNA with a benzaldehyde group hybridized with the complementary guiding DNA, bringing the benzaldehyde group near the amino group of lysine residues on the IgG antibody surface, thereby facilitating conjugation between reactive DNA and IgG antibodies. Finally, complementary DNA liberated the reactive DNA strand and AEC removed unconjugated DNA and proteins, as well as dissociated peptide of guiding DNA.

Using site‐directed azide group labelling, DNA featuring a DBCO group was conjugated to IgG antibodies (Belimumab) (Figure 5c).^[^ ^199^ ^]^ A new lysine‐directed labelling reagent (LDLR) with an azide group was designed, where the salicylaldehyde group of the LDLR can form an iminium ion with lysine residues of IgG antibodies (Belimumab, Cetuximab, Pertuzumab, Trastuzumab, and Rituximab). Adjacent nucleophilic lysines can attack the labile intramolecular ester of the LDLR due to proximity, efficiently inserting an azide group into IgG antibodies via stable covalent bond formation, while the salicylaldehyde iminium ion leaving group undergoes hydrolysis. Subsequently, the azide‐modified antibody, Belimumab, was conjugated to DBCO‐modified DNA using the SPAAC method. Belimumab‐DNA conjugates were purified using AEC, with separation achieved between Belimumab with 1 and 2 conjugated DNA strands. The LDLR reagent for site‐directed labelling reactions was further used to synthesize anti‐HSA (human serum albumin) antibody‐DNA and anti‐human IgG antibody‐DNA (Rituximab, Cetuximab, Trastuzumab,^[^ ^55^ ^]^ and Panitumumab) conjugates,^[^ ^26^ ^]^ which were purified through AEC.

Mouse anti‐PD‐L1 antibodies were conjugated to DNA by first reacting the antibodies with NHS‐DBCO, followed by SEC (MWCO 7 kDa Zeba Spin) to remove unreacted reagent.^[^ ^210^ ^]^ The resulting anti‐PD‐L1‐DBCO was then coupled to azide‐modified DNA, and excess DNA was removed by ultrafiltration (MWCO 50 kDa). In the same study, an alternative enzymatic strategy employed PNGase together with a bifunctional azide‐PEG3‐amine linker to introduce azido groups on anti‐PD‐L1. Excess linker and enzyme were removed by ultrafiltration (30 kDa), after which the azido‐modified antibodies were coupled to DBCO‐functionalized DNA. AEC (Resource Q column) was used to remove residual reactants. In another study, DNA with a thiol group on its 3′‐ and 5′‐ends was conjugated to anti‐CD antibodies (CD3, CD19, CD22, CD28, CD33, CD123, and CD137) through a sulfo‐SMCC linker, followed by purification using AEC.^[^ ^192^ ^]^

Affibodies offer an alternative to antibodies in therapeutic and diagnostic applications.^[^ ^211^ ^]^ They are small non‐immunoglobulin affinity proteins that are engineered through combinatorial protein engineering and selection from numerous variants, identifying those binding to target proteins. To prepare affibody‐DNA conjugates, DNA with an amino group on its 5′‐end was conjugated to the thiol group of cysteine residues on the human epidermal growth factor receptor 2 (HER2) affibody surface using a sulfo‐SMCC linker.^[^ ^206^ ^]^ The affibody is a small protein composed of 58 AAs (MW 6 kDa).^[^ ^212^ ^]^ AEC was used to remove excess HER2 affibody. However, due to the low MW of HER2 affibodies, separating DNA (MW 6 kDa) from HER2 affibody‐DNA conjugates via AEC is challenging. To address this, a Ni‐NTA column was used to remove excess DNA, leveraging the presence of the metal‐binding region (histidine‐rich residues) on HER2 affibodies, followed by dialysis.

#### 3.3.4 Functional Proteins

Other functional proteins encompass a wide range of MWs, spanning from less than 10 kDa to over 200 kDa. This includes engineered proteins such as green fluorescent protein (GFP),^[^ ^213^ ^]^ and naturally occurring proteins like insulin,^[^ ^207^ ^]^ CRISPR (clustered regularly interspaced short palindromic repeats)‐associated protein 9 (Cas9),^[^ ^198^ ^]^ or ovalbumin (OVA).^[^ ^23^ ^]^ Conjugating such proteins to nucleic acids has been explored for applications like SNAs and intracellular delivery.^[^ ^182^, ^198^ ^]^ Common purification methods include AEC^[^ ^183^, ^213^, ^214^, ^215^, ^216^ ^]^ and ultrafiltration,^[^ ^183^, ^184^, ^217^ ^]^ with RP‐HPLC^[^ ^207^ ^]^ also used at times. For instance, to obtain protein‐based DNA‐functionalized Janus particles, the orthogonal reactivity of lysine and cysteine residues has been used. DNA with an amine group was linked to a single surface cysteine residue of enhanced green fluorescent protein mutant (mEGFP) using a SPDP linker.^[^ ^183^ ^]^ Ultrafiltration (MWCO 30 kDa) removed excess DNA, followed by AEC to isolate mEGFP with DNA modifications at the cysteine site from unconjugated mEGFP. Then, the lysine residues of purified mEGFP‐DNA conjugates were further modified with an NHS‐PEG_4_‐N_3_ linker for reaction with DNA with a DBCO group at its 5′‐end to form Janus nanoparticles. Excess DNA was removed by ultrafiltration (MWCO 50 kDa), with SEC then used to obtain purified mEGFP‐DNA conjugates, averaging 14 DNA modifications per mEGFP. Although SEC cannot precisely separate conjugates with different specific numbers of DNA modifications, it was chosen here as it does not interfere with further assembly. Similarly, mEGFP was conjugated to DNA with an amine group for hybridization chain reaction (HCR) studies,^[^ ^214^ ^]^ followed by IMAC purification to remove excess DNA. AEC was performed to separate mEGFP from mEGFP‐DNA conjugates. In a crystallization study, mGFP was conjugated to DNA via a SPDP linker attached to a single surface cysteine residue,^[^ ^213^ ^]^ and excess DNA was removed through IMAC purification. Subsequently, AEC separated the thiol and disulfide forms of mGFP from mGFP‐DNA conjugates.

Mutant selected stable protein 1 (Sp1m) (MW ≈ 300 kDa) has 24 primary amino groups, including surface lysine and N‐termini residues axially, and 12 thiol groups containing surface cysteine residues equatorially. Spatially conjugating DNA to Sp1m allows for hierarchical assembly across one to three dimensions. Initially, a maleimide‐azide linker was attached to equatorial thiol groups of surface cysteine residues of Sp1m, followed by the conjugation of a methyltetrazine‐PEG_5_‐NHS ester linker to axial amine groups of lysine and N‐termini residues. This sequential process installs azide groups equatorially and tetrazine groups axially.^[^ ^184^ ^]^ Subsequently, trans‐cyclooctene (TCO)‐DNA and DBCO‐DNA were conjugated to Sp1m. Ultrafiltration (MWCO 30 kDa) removed most excess DNA, with SEC purification used to remove any remaining DNA. There were ≈10 equatorially conjugated DNA strands and 6 to 8 axially conjugated DNA strands per Sp1m.

CRISPR/Cas9 systems have found broad applications in genome editing. In one study, DNA with a DBCO group was linked to lysine residues of surface Cas9 through an NHS‐PEG4‐azide linker, followed by the formation of Cas9 SNAs. SEC purification removed excess DNA.^[^ ^198^ ^]^ There were 14 conjugated DNA strands per Cas9 protein. In another study, DNA with an amino group at its 5′‐end was conjugated to the thiol group of cysteine residues of OVA through LPFC via an SMCC linker. This conjugate was incorporated into DNA origami for immune stimulation, with ultrafiltration (MWCO 30 kDa) used to remove excess DNA.^[^ ^23^ ^]^ Additionally, DNA with an azide group was coupled to the amino group of lysine‐29 of the B chain (B29 lysine) residue of insulin (MW 5.8 kDa) via a DBCO‐sulfo‐NHS linker for hybridization with DNA origami in intracellular studies (Figure 5d).^[^ ^207^ ^]^ The reaction mixture underwent RP‐HPLC purification (C18, gradient: 27–45% ACN in TEAA) to remove excess DNA and unconjugated insulin, followed by concentration through ultrafiltration (MWCO 3 kDa).

### 3.4 Lipid‐Nucleic Acid Conjugates

Conjugates of nucleic acids with lipid molecules serve various purposes, including modifying liposomes,^[^ ^77^ ^]^ mediating vesicle fusion,^[^ ^218^ ^]^ anchoring DNA nanostructures to membranes,^[^ ^219^ ^]^ facilitating drug delivery,^[^ ^11^, ^220^ ^]^ modulating membrane organization,^[^ ^27^ ^]^ and studying structure formation.^[^ ^221^, ^222^ ^]^ The hydrophilic nucleic acid domain provides structural programmability and addressability, while the hydrophobic lipid domain enables membrane insertion and interaction with lipophilic environments. This amphiphilic duality underlies both the functional utility and the experimental challenges of these conjugates, particularly regarding solubility, aggregation, and purification.

Purification of lipid‐nucleic acid conjugates primarily relies on RP‐HPLC, which separates species based on hydrophobicity differences introduced by the lipid moiety.^[^ ^221^, ^223^ ^]^ The choice of stationary phase (commonly C18 or C4), organic solvent gradient (usually ACN with ion‐pairing agents such as TEAA), and temperature must be carefully optimized to avoid aggregation or incomplete elution. RP‐HPLC provides excellent resolution between unmodified oligonucleotides, partially lipidated intermediates, and fully conjugated products, but the strong hydrophobic interactions of these amphiphiles can lead to peak broadening or poor recovery if gradient slopes or solvent strengths are not properly tuned. For very hydrophobic conjugates, shallower gradients or less retentive C4 columns are preferred, whereas highly polar conjugates may require stronger gradients or alternative ion‐pair reagents.

Two main synthetic routes are used: stepwise solid‐phase (presynthetic) and solution‐phase (postsynthetic) coupling.^[^ ^13^ ^]^ Solid‐phase approaches, often implemented during automated oligonucleotide synthesis, simplify purification by allowing intermediate washing steps to remove unreacted reagents or by‐products directly on the solid support.^[^ ^13^, ^218^, ^224^ ^]^ Cleavage from the support then yields conjugates that can be subjected to final purification by RP‐HPLC under aqueous/organic gradients. In contrast, solution‐phase reactions, demand careful control of solvent polarity and micelle formation. Excess hydrophobic reagents can precipitate or form aggregates that complicate downstream separation. RP‐HPLC remains the method of choice for final purification, often complemented by ultrafiltration to remove low‐molecular‐weight reagents or byproducts.^[^ ^225^, ^226^ ^]^

**Figure 6:**
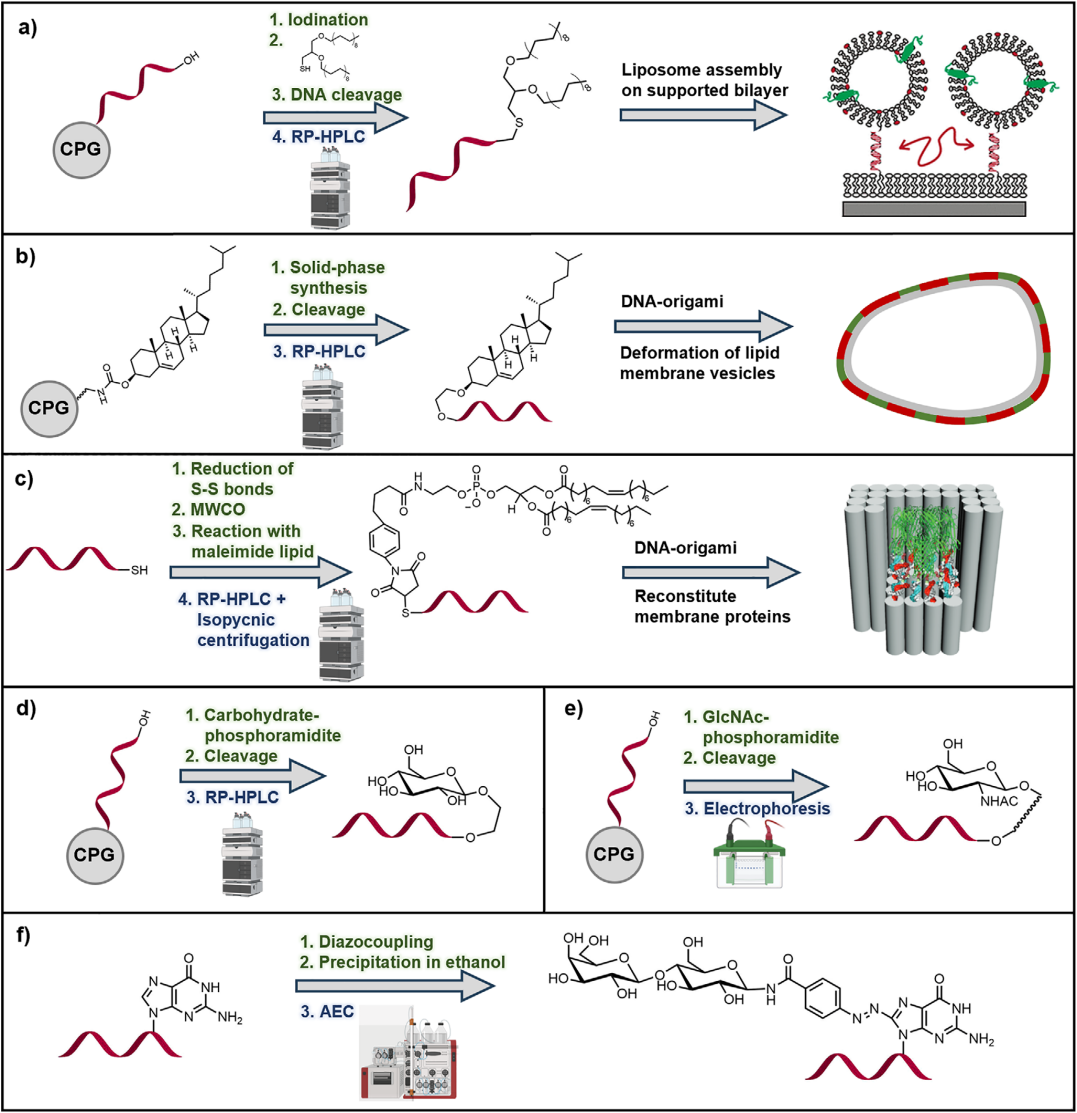
Synthesis and purification strategies of lipid‐DNA and saccharide‐DNA conjugates. a) DNA oligonucleotides are functionalized with (C18)_2_ lipids by iodination of 5′‐end, followed by reaction with lipid‐thiolate. RP‐HPLC was used to remove unreacted and iodinated oligonucleotides. Parts reproduced with permission.^[^ ^77^ ^]^ Copyright 2005, American Chemical Society. b) A 3′‐cholesteryl‐TEG CPG was used to introduce a cholesterol modification at the 3′‐end of DNA oligonucleotides, which was purified by RP‐HPLC.^[^ ^27^ ^]^ c) DNA oligonucleotides with thiol ends were reduced, followed by MWCO filtration, and functionalized with maleimide lipids. The conjugates were purified by RP‐HPLC. Parts reproduced with permission.^[^ ^225^ ^]^ Copyright 2018, John Wiley and Sons. d) Carbohydrate‐DNA conjugates achieved by phosphoramidite chemistry and purification using RP‐HPLC.^[^ ^28^ ^]^ e) Synthesis of carbohydrate‐DNA conjugates by phosphoramidite chemistry, followed by dPAGE purification.^[^ ^78^ ^]^ f) DNA and RNA were functionalized with lactose and cellobiose at the guanine bases by diazo coupling of carbohydrate diazonium salts. The conjugates were precipitated into ethanol, collected by centrifugation and purified by AEC.^[^ ^234^ ^]^ Synthesis and purification steps are highlighted in green and blue, respectively, for clarity. (Figure created with BioRender.com).

For example, DNA oligonucleotides were functionalized with (C_18_)_2_ lipids by removing the terminal dimethoxytrityl (DMT) group at the DNA 5′‐end during oligonucleotide synthesis, followed by iodination to make the 5′‐end electrophilic, reaction with lipid‐thiolate, and final deprotection and cleavage from the solid support (**Figure** **6**a).^[^ ^77^, ^227^ ^]^ Purification involved separating unreacted and iodinated oligonucleotides from the lipid‐oligonucleotide conjugates using RP‐HPLC (C4 column) with a gradient of 0–60% ACN in TEAA buffer.^[^ ^77^ ^]^

In an alternative approach, phosphoramidite chemistry was used to modify DNA oligonucleotides at both the 3′‐ and 5′‐end.^[^ ^218^, ^224^, ^228^, ^229^, ^230^, ^231^ ^]^ (C_18_)_2_ lipids were functionalized with phosphoramidite groups and coupled to the DNA oligomer as the last “base” using a DNA synthesizer. RP‐HPLC purification was then performed.^[^ ^218^ ^]^ To modify siRNA with various lipids, phosphoramidite chemistry was used as well, followed by RP‐HPLC purification (Source 15RPC column) with a 10–70% gradient of ACN in sodium acetate.^[^ ^35^ ^]^ In another study, a cholesterol modification was introduced at the 3′‐end of DNA oligonucleotides using commercial 3′‐cholesteryl‐TEG (triethylene glycol spacer) CPGs during oligonucleotide synthesis (Figure 6b).^[^ ^27^ ^]^ The functionalized oligonucleotides were purified using RP‐HPLC (C18 column).^[^ ^27^ ^]^ Similarly, DNA was functionalized at both the 3′‐ and 5′‐end using commercially obtained oligonucleotides with thiol end (Figure 6c).^[^ ^225^ ^]^ After reducing disulfide bonds in the modified DNA, followed by ultrafiltration (3 kDa MWCO), a maleimide lipid was added and the product was purified by RP‐HPLC (C4 column) using a gradient of 10–40% ACN.^[^ ^225^ ^]^ In some cases, lipid‐DNA conjugates, which were incorporated into liposomes, were separated from unconjugated DNA using isopycnic centrifugation in iodixanol gradients (which separates species based on buoyant density),^[^ ^232^ ^]^ or purified on agarose gel in the presence of Triton X‐100 while HPLC or dPAGE could not be used due to micelle formation.^[^ ^233^ ^]^

For solution‐phase coupling, squalene was coupled to the 3′‐end of siRNA via maleimide‐sulfhydryl chemistry, and excess squalenoyl maleimide was washed away. Purification was monitored using RP‐HPLC.^[^ ^226^ ^]^ The choice of RP‐HPLC protocol depends on lipid polarity, chain length and the eluents used. C18 columns are suitable for non‐polar samples, while C4 columns offer versatility, especially for very highly hydrophobic conjugates, and with high ACN gradients.

### 3.5 Saccharide‐Nucleic Acid Conjugates

Conjugates of nucleic acids with saccharides or carbohydrates represent an important class of biohybrid molecules that combine the programmability of nucleic acids with the biocompatibility and recognition properties of sugars. These conjugates have been explored for enhancing the stability and cellular uptake of DNA and RNA constructs,^[^ ^28^, ^235^ ^]^ exploiting carbohydrate‐mediated recognition at cellular membranes. Furthermore, DNA has been conjugated to the surface of living cells by engineering oligosaccharides on the cell surface via incorporating azides which enable DNA linkage.^[^ ^236^ ^]^

Unlike amphiphilic or hydrophobic conjugates, saccharide‐modified oligonucleotides typically exhibit minimal retention on reversed‐phase materials and may display overlapping elution with unreacted oligonucleotides. As a result, purification requires shallow gradients and the use of ion‐pairing agents to achieve adequate separation. RP‐HPLC is the most widely used technique, often employing C18 columns and mild gradients of ACN in TEAA buffer.^[^ ^28^, ^235^, ^237^, ^238^, ^239^ ^]^

Several coupling strategies are employed depending on whether the carbohydrate is introduced during solid‐phase synthesis or postsynthetically in solution. Solid‐phase methods using phosphoramidite chemistry are advantageous because intermediate washing steps efficiently remove excess reagents, facilitating high‐purity conjugates upon cleavage from the support. For instance, it allows functionalizing the DNA 5′‐end with saccharides such as glucose, fucose, maltose, and maltotriose in a DNA synthesizer.^[^ ^28^ ^]^ These saccharide‐DNA conjugates can be purified using RP‐HPLC (C18 column) with a gradient of 5–37.5% ACN (Figure 6d).^[^ ^28^ ^]^ Similarly, DNA was functionalized on solid support at both the 3′‐ and 5′‐ends with carbohydrates^[^ ^240^, ^241^ ^]^ like methyl 4′‐deoxy‐lactoside,^[^ ^242^ ^]^ with purification achieved by dPAGE (Figure 6e).^[^ ^78^, ^242^ ^]^ Oligoribonucleotide conjugates with glucose or galactose at the RNA 5′‐end were synthesized using phosphoramidite chemistry and purified by RP‐HPLC (C18 column).^[^ ^237^ ^]^

In another method, DNA was functionalized at the 5′‐end using SPFC.^[^ ^235^ ^]^ The oligonucleotide was subjected to carbonyldiimidazole (CDI), ethylenediamine and hexamethylene‐1,6‐disocyanate to yield the isocyanato derivative, which was then reacted with the amino sugars d‐glucosamine and d‐galactosamine. These saccharide‐DNA conjugates were purified using RP‐HPLC.^[^ ^235^ ^]^ DNA oligonucleotides were also functionalized with carbohydrates using 3,4‐diethoxy‐3‐cyclobutene‐1,2‐dione as a linking reagent between carbohydrates with amino group at the reducing end and DNA with aminoalkyl modification, with purification achieved by RP‐HPLC (C18 column).^[^ ^238^ ^]^ A similar approach was followed to prepare mannosylated oligoribonucleotides.^[^ ^243^ ^]^ Carbohydrate conjugation to DNA and RNA was also achieved by diazo coupling of carbohydrate diazonium salts to the guanine bases, with purification through precipitation into ethanol followed by centrifugation and AEC (Figure 6f).^[^ ^234^, ^244^ ^]^ In another method, aldehyde‐containing DNA was conjugated to the aminooxy group at the reducing end of a saccharide, followed by RP‐HPLC purification.^[^ ^245^ ^]^ Additionally, carbohydrates with an alkylamine linker were attached to NHS‐carboxy‐dT phosphoramidite during DNA synthesis, with purification by RP‐HPLC (C18 column) with a 6–12% ACN gradient in TEAA buffer.^[^ ^239^ ^]^ Overall, most saccharide‐nucleic acids conjugates were purified by RP‐HPLC using a C18 column with a gradient of low amounts of ACN (5–37.5%).

## 4 Conclusion and Outlook

The synthesis and purification of nucleic acid conjugates are central to advances in biotechnology, medicine, and nanotechnology. This review has outlined the main strategies for preparing and purifying diverse classes of nucleic acid conjugates, including polymer‐, peptide‐ and protein‐, lipid‐, and saccharide‐nucleic acid hybrids. Each class poses distinct chemical and physical challenges, requiring tailored purification approaches. The choice of method depends strongly on the conjugate's molecular characteristics, such as charge, hydrophobicity, and size, as well as on the desired application and available infrastructure. Polymer‐nucleic acid conjugates are vital for creating robust, biocompatible materials for drug delivery, hydrogels, and biosensors. Peptide and protein‐nucleic acid conjugates are essential for therapeutic applications and diagnostics. Lipid‐nucleic acid conjugates leverage amphiphilicity for drug delivery and membrane studies, while saccharide‐nucleic acid conjugates enhance the stability and cellular uptake of nucleic acids through carbohydrate recognition.

Future work in nucleic acid bioconjugation will likely integrate automated synthesis platforms with advanced, scalable purification workflows such as multi‐modal chromatography, microfluidic purification, and machine‐learning assisted optimization, thus enhance the efficiency and specificity of conjugation reactions and refined purification. Developments in chromatography optimization, new affinity tags, and hybrid purification strategies are expected to achieve higher purity and yield. Addressing purification scalability and reproducibility as well as green‐chemistry aspects will be critical for industrial and clinical applications.

Beyond yield, purity and scalability, analytical characterization and process control will become increasingly critical. As conjugates move toward clinical applications, the ability to determine stoichiometry, residual unconjugated species, aggregation state, and functionality rapidly and robustly will dictate their success to meet regulatory and manufacturing quality requirements. While the chemistry of nucleic acid conjugation is mature in many respects, the downstream purification, analytical control and manufacturing readiness represent challenges for enabling widespread, robust application of these hybrid biomaterials.

## Conflict of Interest

The authors declare no conflict of interest.
